# Single-cell RNA sequencing reveals rebalancing of immunological response in patients with periodontitis after non-surgical periodontal therapy

**DOI:** 10.1186/s12967-022-03702-2

**Published:** 2022-11-03

**Authors:** Hansong Lee, Ji-Young Joo, Dong Hyun Sohn, Junho Kang, Yeuni Yu, Hae Ryoun Park, Yun Hak Kim

**Affiliations:** 1 Convergence Medical Sciences, Pusan National University, 50612 Yangsan, Republic of Korea; 2 Department of Periodontology, School of Dentistry, Pusan National University, 50612 Yangsan, Republic of Korea; 3 Department of Microbiology and Immunology, School of Medicine, Pusan National University, 50612 Yangsan, Republic of Korea; 4 Medical Research Institute, Pusan National University, 50612 Yangsan, Republic of Korea; 5 Department of Oral Pathology, School of Dentistry, Pusan National University, 49 Busandaehak- ro, 50612 Yangsan, Republic of Korea; 6 Department of Anatomy, School of Medicine, Pusan National University, 49 Busandaehak-ro, 50612 Yangsan, Republic of Korea

**Keywords:** Chronic periodontitis, Immune response, Single-cell RNA sequencing, Therapeutic target, CRIP1, RESISTIN

## Abstract

**Background:**

Periodontitis is a major inflammatory disease of the oral mucosa that is not limited to the oral cavity but also has systemic consequences. Although the importance of chronic periodontitis has been emphasized, the systemic immune response induced by periodontitis and its therapeutic effects remain elusive. Here, we report the transcriptomes of peripheral blood mononuclear cells (PBMCs) from patients with periodontitis.

**Methods:**

Using single-cell RNA sequencing, we profiled PBMCs from healthy controls and paired pre- and post-treatment patients with periodontitis. We extracted differentially expressed genes and biological pathways for each cell type and calculated activity scores reflecting cellular characteristics. Intercellular crosstalk was classified into therapy-responsive and -nonresponsive pathways.

**Results:**

We analyzed pan-cellular differentially expressed genes caused by periodontitis and found that most cell types showed a significant increase in *CRIP1*, which was further supported by the increased levels of plasma CRIP1 observed in patients with periodontitis. In addition, activated cell type-specific ligand-receptor interactions, including the BTLA, IFN-γ, and RESISTIN pathways, were prominent in patients with periodontitis. Both the BTLA and IFN-γ pathways returned to similar levels in healthy controls after periodontal therapy, whereas the RESISTIN pathway was still activated even after therapy.

**Conclusion:**

These data collectively provide insights into the transcriptome changes and molecular interactions that are responsive to periodontal treatment. We identified periodontitis-specific systemic inflammatory indicators and suggest unresolved signals of non-surgical therapy as future therapeutic targets.

**Supplementary Information:**

The online version contains supplementary material available at 10.1186/s12967-022-03702-2.

## Background

Periodontitis is a highly prevalent disease in humans and is the sixth most common human disease [1]. As reclassified at the World Workshop in 2017, periodontitis has a clear distinction from gingivitis in that periodontitis manifests as a loss of alveolar bone and periodontal tissue and has permanent bone defects even after periodontal therapy [1, 2]. In the past, periodontitis has been suggested to be a simple inflammatory disease in the periodontium that is induced by providing a habitat for microbes. However, it is now defined as a complex disease induced by repeated interactions between host modulation of inflammatory and immune activities, periodontal pathogens, and environmental factors [3]. Thus, the susceptibilities to periodontitis are determined by the host response, specifically the magnitude of the inflammatory response and the differential activation of immune pathways rather than the amount and/or the pathogenicity of periodontal bacteria [4, 5].

Although periodontitis is an inflammatory disease localized to the oral cavity, it has been known to be associated with various systemic diseases, such as cardiovascular diseases, diabetes mellitus, and rheumatoid arthritis [6, 7]. Several possibilities have been proposed for the mechanism by which local tissues exert systemic effects. The surface of ulcerated periodontal pockets may allow microbial products, such as lipopolysaccharide (LPS) or proteases, to enter the circulation, resulting in bacteremia [7]. In addition, patients with severe periodontitis have elevated levels of inflammatory mediators, such as IL-1, IL-6, C-reactive protein (CRP), and fibrinogen, in their blood [8–10]. Thus, periodontitis can affect the whole body and induce a systemic inflammatory response through leakage of microbial products into the circulatory system [7, 11]. Therefore, treatment for periodontitis is fundamental not only to relieve immediate pain, but also to attenuate the subsequent systemic response.

Recent advances in single-cell RNA sequencing (scRNA-seq) technology have enabled us to understand and elucidate cellular complexities [12]. Since each cell type, especially immune cells, has unique functions, pathways, and regulatory mechanisms, it is essential to characterize cell type-specific genes to identify disease biomarkers and therapeutic targets, which is feasible using scRNA-seq technology. To examine the main factors for chronic inflammation induced by periodontitis, we compared the expression profiles of peripheral blood mononuclear cells (PBMCs) from patients with periodontitis and healthy controls. In addition, to determine whether therapeutic intervention can redirect the immunologic status of periodontitis, we delineated therapy-responsive and -nonresponsive intercellular pathways.

## Methods

### Sample preparation and inclusion criteria

Participants for scRNA-seq analysis and enzyme-linked immunosorbent assay (ELISA) were recruited from the Department of Periodontology, Pusan National University Dental Hospital, under protocols approved by the Institutional Review Board (IRB no. PNUDH-2020-001, and IRB no. PNUDH-2020-032 for scRNA-seq analysis and ELISA, respectively). All participants provided written informed consent. All patients diagnosed with periodontitis stage III were included in this study, and the severity of periodontitis was defined according to the 2017 World Workshop criteria, a new classification scheme for periodontal and peri-implant diseases and conditions [2]. Healthy periodontal status was defined as a pocket depth ≤ 3 mm and no bleeding on probing, with no signs of clinical inflammation, including redness and swelling. The inclusion criteria were subjects with 10 or more teeth and no systemic diseases. In addition, we excluded subjects who had received periodontal treatment within the last 6 months or anti-inflammatory drugs or antibiotics within the last 6 weeks. Those who were previously or currently heavy smokers (which we defined as people who smoked more than one pack per day) were also excluded. All participants with periodontitis received non-surgical periodontal treatment from the same periodontist. Non-surgical periodontal treatment consisted of full-mouth scaling and root planning (SRP) performed in a single appointment under local anesthesia using an ultrasonic device and hand instruments. We attempted to minimize confounding factors that could arise between treatments. Oral hygiene instruction was provided and consisted of the Bass brushing technique and use of an interdental brush and dental floss. The participants were instructed not to use any antimicrobial mouth-rinsing solutions for the duration of the study. The periodontal clinical parameters before and after one month of treatment, as well as the demographic features, are presented in Additional file 1: Table S1.

### Blood collection and PBMC isolation

Peripheral blood samples were collected in plastic blood collection tubes containing EDTA. PBMCs for scRNA-seq were isolated using SepMate (Stemcell Technologies Inc.) within 30 min of collection according to the manufacturer’s instructions. Briefly, density gradient medium and diluted blood samples were added to a SepMate tube. After carefully mixing the medium and samples, the tubes were centrifuged at 1200 × *g* for 10 min. The top layers were poured into a new tube and washed twice with phosphate-buffered saline containing 2% fetal bovine serum. The tubes were then centrifuged at 300 × *g* for 8 min at room temperature. The collected PBMCs were frozen and stored at − 80 °C until sequencing. Plasma for ELISA was collected after centrifugation at 2,000 × *g* for 10 min at room temperature and stored at − 80 °C until use.

### Enzyme-linked immunosorbent assay (ELISA)

The blood samples in EDTA were centrifuged at 2,000 × *g* for 10 min at 4 °C, and the supernatant was stored at − 70 °C until analysis. For the measurement of TNF-α, IFITM1 and CRIP1 concentrations, Human TNF-α Uncoated ELISA (Invitrogen, Vienna, Austria), Human Interferon Induced Transmembrane Protein 1 (IFITM1) ELISA (MyBioSource, Vancouver, Canada) and Cysteine-rich protein 1 ELISA (Mybiosoruce, San Diego, CA, USA) kits were used, respectively. Briefly, plasma samples were added to assay plates pre-coated with anti-TNF-α, IFITM1 or CRIP1 antibodies and incubated for 2 h. The plates were then incubated with diluted detection antibody for 1 h, reacted with the substrate solution for 30 min, followed by the addition of the stop solution. All reactions for TNF-α, IFITM1 and CRIP1 were performed at RT and 37 °C, respectively. Standard curves were plotted as control versus the mean optical density at 450 nm. TNFα, IFITM1 and CRIP1 concentrations in each sample were quantified based on a standard curve. Clinical information about the participants whose plasma was used for ELISA is shown in Additional files 2, 3: Tables S2, S3.

### Library preparation and sequencing of scRNA

Libraries were prepared using the chromium controller according to the 10× chromium Next GEM Single Cell 3ʹ v3.1 protocol. The cell suspension was mixed with the master mix and loaded with Single Cell 3ʹ v3.1 Gel Beads and Partitioning Oil into a chromium Next GEM chip G. RNA transcripts from single cells were uniquely barcoded and reverse-transcribed within droplets. cDNA molecules were pooled and then subjected to end repair, addition of a single ‘A’ base, and ligation of the adapters. Next, the products were purified and enriched using PCR to create a final cDNA library. Finally, the libraries were sequenced using the Illumina HiSeq platform according to the read length provided in the user guide.

### scRNA-seq data pre-processing

Single-cell gene expression data were processed using 10× Genomics Cell Ranger v3.1.0. Raw BCL files from the Illumina sequencing platform were demultiplexed to generate FASTQ files using the ‘cellranger mkfastq’ pipeline. Then, raw FASTQ files were analyzed using the ‘cellranger count’ pipeline. This step includes alignment to the human reference genome (GRCh38, v3.0.0) and measurement of gene expression with a unique molecular identifier (UMI) and cell barcode. Consequently, a cell-by-gene count matrix was generated. To remove low-quality cells, cells with less than 500 UMIs or more than 20,000 UMIs and > 20% mitochondrial genes were filtered out. In addition, we removed cells with fewer than 250 genes or more than 5000 genes, as well as cells with less than 80% complexity (number of genes detected per UMI with log transformation), which could be interpreted as specific cell types, artifacts, or contaminants. In addition, we included genes expressed in more than 0.1% of the cells, not only to eliminate zero counts, but also to prevent genes expressed in a few cells from lowering the average of all other cells. As some samples had a large number of cells (maximum 12,177 cells), possible doublets were estimated using Scrublet, and 3.5% of cells were eliminated (maximum 11,852 cells) [13].

### Analysis of scRNA-seq data

The Seurat (v3.2.2) R package was used to integrate, scale, cluster, and visualize data. The remaining count data were normalized using the SCTransform function based on regularized negative binomial regression on total cellular read counts [14, 15]. After normalization of each sample, the FindIntegrationAnchors and IntegrateData functions were used for integration with the largest dataset among the 12 samples, which was used as the reference dataset. Then, scaling and principal component analysis (PCA) were performed using the ScaleData and RunPCA functions, and the first 30 principal components that were selected depending on the elbow plot were utilized to construct the UMAP dimension reduction and shared nearest-neighbor graph (SNN) using RunUMAP and the FindNeighbors function. Then, cell clusters were distinguished using the graph-based modularity optimization algorithm of the Louvain method for detecting communities with a setting resolution of 0.6, which is able to clearly specify the cell type as well as detect tiny molecular signals [16]; ultimately, 26 clusters were differentiated. Cell identity markers were identified using the FindAllMarkers function, and genes with a log-fold change threshold > 0.5 and false discovery rate (FDR) < 0.01 were regarded as significant differentially expressed genes (DEGs).

To annotate each cluster into immune cell types, these cell identity markers were supplied into the SingleR (v3.12) R package [17]. SingleR first assigned a single cell into highly matched cell types and then annotated and combined clusters into the most likely cell types. Most of the cells in the same cluster were suggested to be of consistent cell type, usually over 70%, whereas clusters with low consistency were annotated using multiple genes representing cell-specific signatures.

DEG analysis was conducted using edgeR and flexible zero-inflated negative binomial-based wanted variation extraction (ZINB-WaVE), which accounts for zero inflation and over-dispersion [18, 19]. To identify periodontitis-related genes, we compared the expression differences between the three groups as follows: (1) healthy control vs. pre-treatment and (2) pre-treatment vs. post-treatment. In case (2), we compared gene expression with a paired test. DEGs with log2FC > 0.3 and FDR value < 0.05, were extracted and log2FC was recalculated to compare chronic periodontitis status with that of the other two groups.

### Gene ontology (GO) analysis

To investigate the biological function of the DEGs detected in the pre-treatment group, we used the R package ‘clusterProfiler’ (ver. 4.0.5) [20]. The upregulated or downregulated genes in each cell type were used to determine the biological function, and the gene set with *p* < 0.05 and more than one enriched gene was considered to be significant. For genes with more than five biological functions, the top five, in the order of the lowest *p*-value, were extracted.

### Cell–cell interaction

We inferred cell‒cell communication based on the expression of ligand-receptor pairs from the manually curated human database CellChatDB. We followed the official workflow for the R package ‘CellChat’ [21]. We created normalized count data from the Seurat object to the CellChat object and pre-processed with the identifyOverExpressedGenes, identifyOverExpressedInteractions, and projectData functions. We generated a ligand‒receptor interaction database comprising secreted signaling, extracellular matrix-receptor, and cell‒cell contact interactions. Then, we calculated the communication probability and inferred cellular communication networks with the computeCommunProb and computeCommunProbPathway functions. Finally, we aggregated the inferred signaling pathways, compared their strength and cellular components, and extracted statistically significant pathways with greater than 1.5-fold signal intensity between the two groups.

### Statistical analyses

Statistical analyses were performed using R version 4.0.3. Kruskal‒Wallis and Wilcoxon rank-sum tests were used to compare cellular composition by setting the significance level at 0.05. PCA plots for clinical information were obtained using the prcomp() function. Receiver operating characteristic (ROC) curves were plotted using the R package ‘ROCit’ [22].

## Results

### Single-cell transcriptional landscape of the PBMCs from patients with periodontitis

**Fig. 1:**
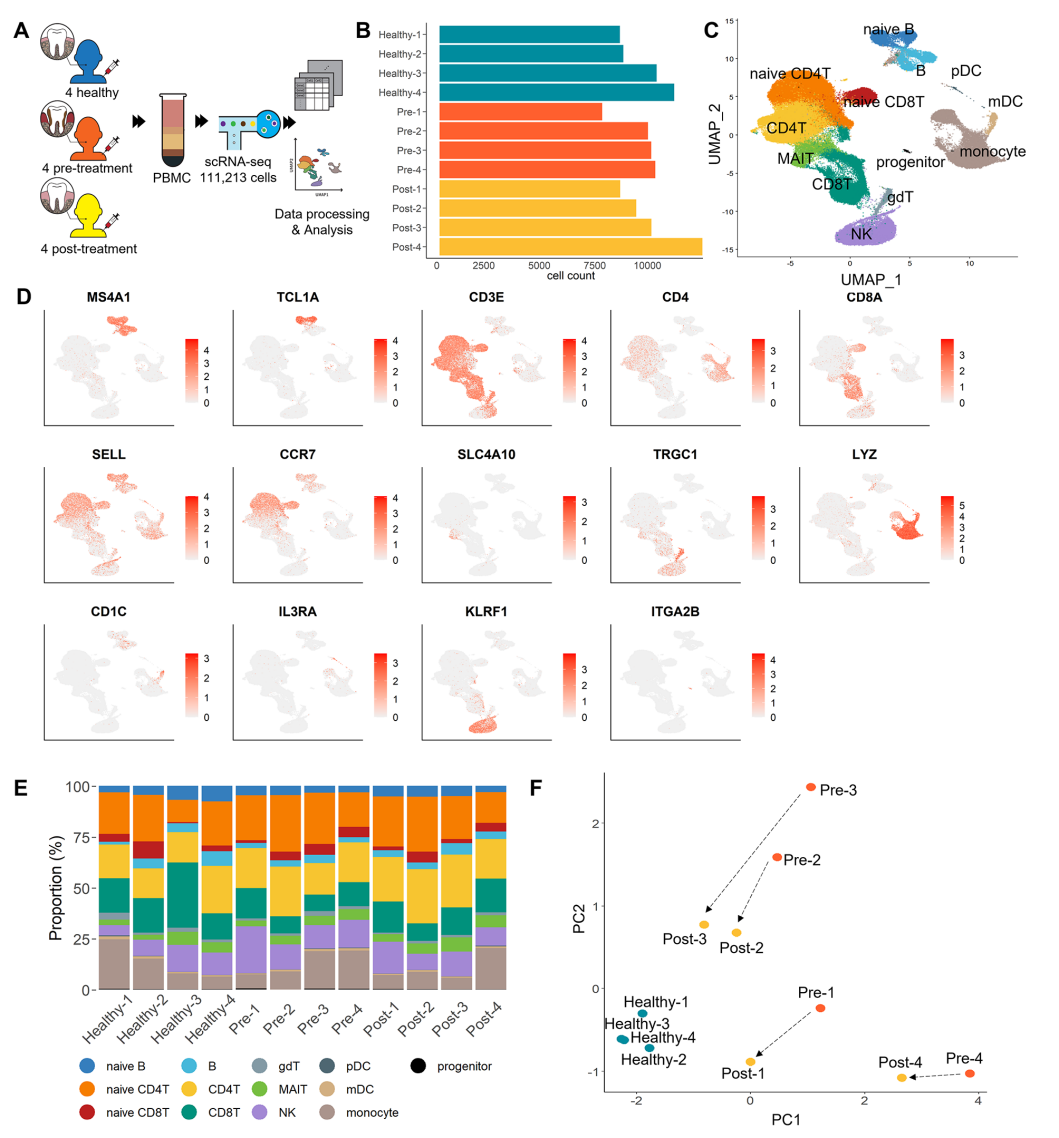
**Composition of peripheral blood mononuclear cells (PBMCs) from healthy donors and pre- and post-treatment periodontitis patients** **A.** The graphical abstract of this study **B.** Bar graph showing cell numbers in each sample. Blue represents healthy donor samples, orange represents pre-treatment patient samples, and yellow represents post-treatment patient samples **C.** UMAP plot of 111,213 PBMCs from all subjects, colored according to the major cell lineages **D.** Scatter plot of canonical marker genes for 13 major lineages projected onto the UMAP plot. The red and gray spectra indicate the expression levels of each gene **E.** Bar graph showing the proportion of the major cell types for each participant, colored according to the cell type **F.** PCA plot of the participants using six clinical variables: ESR, CRP, clinical attachment level, probing pocket depth, plaque index, and gingival index

We isolated PBMCs from four healthy donors and four paired pre-and post-treatment patients for scRNA-seq and obtained 111,213 cells, with an average of 9,268 cells per participant (Fig. 1 A, B). Distinct cell populations were observed after dimensionality reduction and graph-based clustering.

We assigned clusters to the cell type with the highest probability and confirmed the identities using known markers (Fig. 1 C, D). We found 13 major cell compartments that are important for the immune response, and each donor’s PBMCs contained all of these clusters (Fig. 1E). The PBMC populations comprised T cells (60–73%), B cells (4–15%), natural killer (NK) cells (5–23%), monocytes (5–24%), and dendritic cells (1–2%) with normal distributions, regardless of the presence of disease or chronic inflammation-resolved status via periodontal therapy. Although most of the samples showed no significant differences in cellular composition among the three groups, in patients with periodontitis the proportion of CD8^+^ T cells was slightly lower, while naïve CD8T cells were slightly higher compared to those in healthy controls, but these changes were partially rescued after therapeutic intervention (*p =* 0.062 and *p* =, respectively), (Additional file 4: Fig. S1A, B). To verify the clinical inflammatory status of the patients with periodontitis and resolution by periodontal therapy, we performed PCA using six variables: erythrocyte sedimentation rate (ESR) and CRP, which are indicators of inflammation; probing pocket depth (PPD), clinical attachment level (CAL), plaque index (PI), and gingival index (GI), which are indicators of the severity of periodontitis. The healthy controls clustered together but were separated from the pre- and post-treatment groups (Fig. 1 F and Additional file 5: Fig. S2). The pre-treatment group was distinct from the healthy group, while the post-treatment group was more similar to the controls, although the clinical variables of post-treated patients were not comparable to those of the healthy controls. This shows that therapeutic intervention improved the inflammatory status and altered the clinical characteristics of periodontitis.

### Gene expression alterations in innate immune cells of patients with chronic periodontitis

**Fig. 2:**
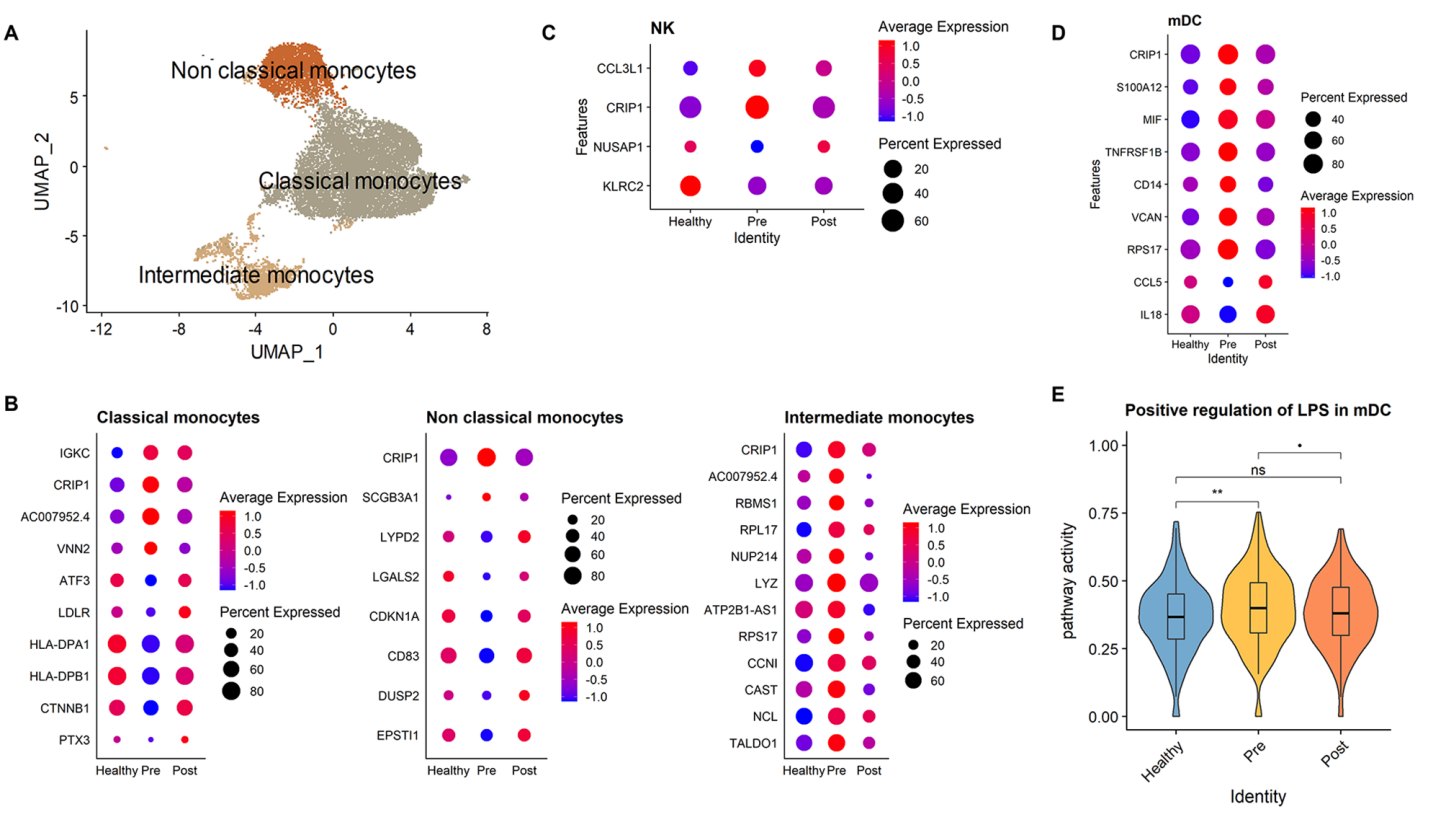
**Differentially expressed genes in innate immune cells, monocytes, dendritic cells, and NK cells** **A.** UMAP plot of the three monocyte subsets: classical, non-classical, and intermediate monocytes **B–D.** Dot plot showing differential expression levels of genes in each monocyte subtype **(B)**, NK cells **(C)** and mDCs **(D)**. Displayed genes showed similar expression in control and post-treatment groups, but inverse expression patterns in the pre-treatment group. The color of the dots represents the expression levels of the gene, whereas dot size represents the percent of cells expressing the gene **E.** Activity score of positive regulation of lipopolysaccharide-mediated signaling pathway in mDCs. Enrichment of genes with GO term GO:0031666 was calculated using the Wilcoxon rank-sum test. p ≤ 0.1 (•), p ≤ 0.05 (*), p ≤ 0.01 (**), p ≤ 0.001 (***), p ≤ 0.0001 (****), p ≥ 0.05 (ns)

Next, we investigated the transcriptome changes in innate immune cells, including monocytes, dendritic cells (DCs), and NK cells. A subtype of DCs, called plasmacytoid dendritic cells (pDCs), which are responsible for antiviral immunity, were excluded as they had no detectable transcriptional changes. The monocytes obtained from Fig. 1 C were re-clustered and classified into three subtypes: classical, non-classical, and intermediate monocytes (Fig. 2 A). The identities of the sub-clusters were determined based on the expression of CD14 and FCGR3A (Additional file 6: Fig. S3A). The DEGs in innate immune cells are shown in Fig. 2B‒D. An increase in cysteine-rich protein 1 (*CRIP1*) in patients with periodontitis is observed in all types of innate immune cells, and *CRIP1* levels after therapy are similar to that in healthy controls, suggesting that CRIP1 may be an important factor in maintaining chronic inflammation. When GO terms were investigated using pre-treatment group-specific DEGs, classical monocytes had low levels of MHC protein complex assembly genes and reduced antigen-presenting activity. In addition, the lymphocyte-activating activity was reduced in non-classical monocytes (Additional file 7: Fig. S4). However, genes responding to LPS stimulation were increased in myeloid DCs (mDCs) of the pre-treatment groups and approached intensity levels similar to the controls following treatment (Fig. 2E, Additional file 8: Fig. S5). These results imply that although monocytes had reduced antigen-presenting function, mDCs actively responded after detection of LPS, a virulence factor of the periodontitis bacteria *Porphyromonas gingivalis* and could be rescued by periodontal therapy [23].

### Systemic properties of adaptive immune cells found in patients with chronic periodontitis

**Fig. 3:**
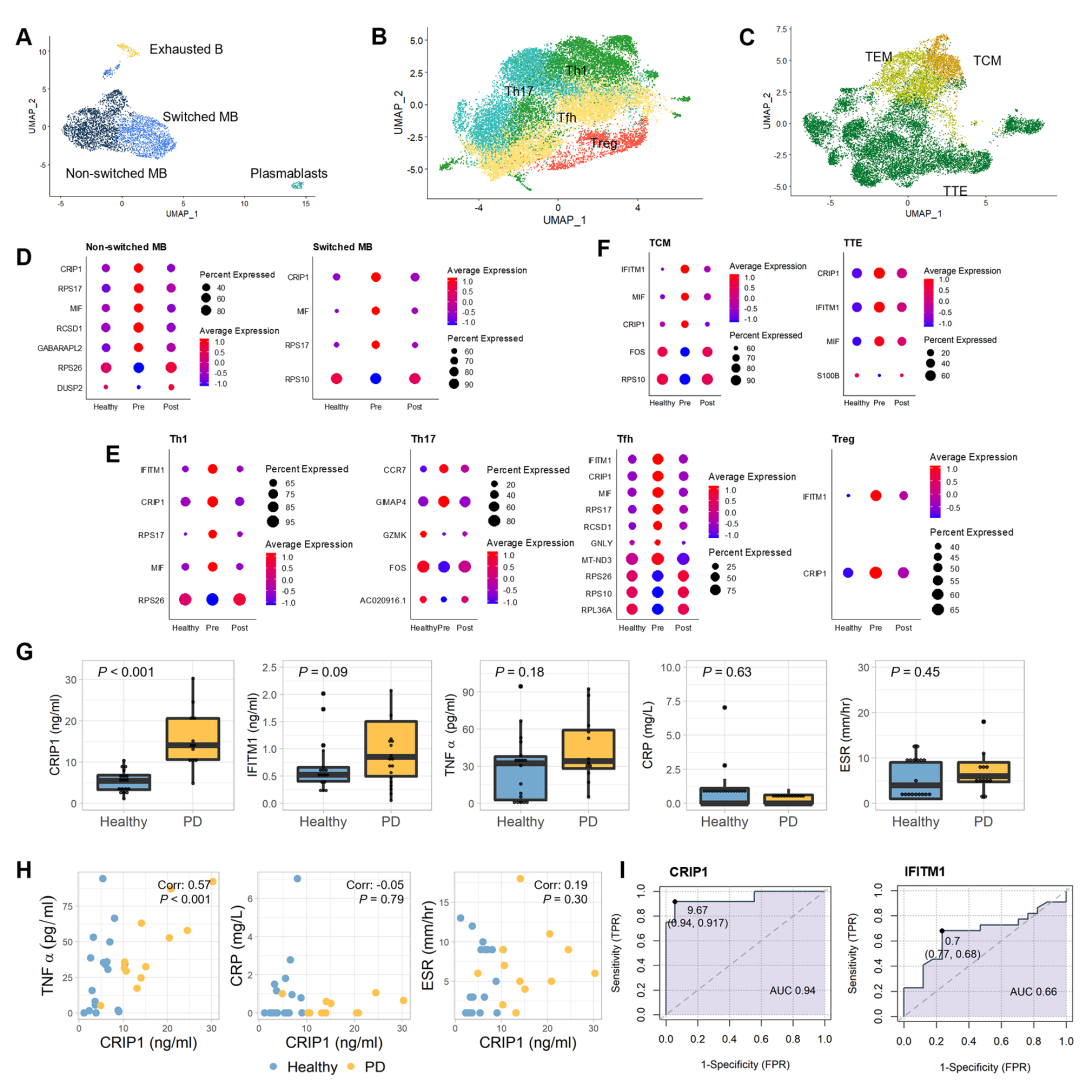
**Periodontitis-induced transcriptional alterations in adaptive immune, B, CD4T, and CD8T cells** **A.** UMAP plot of the four B cell subsets: exhausted B cells, switched memory B (switched MB) cells, non-switched memory B (non-switched MB) cells, and plasmablasts **B.** UMAP plot of the four CD4^+^ T-cell subsets: T helper 1 (Th1), T helper 17 (Th17), follicular helper T (Tfh), and regulatory T (Treg) **C.** UMAP plot of the three CD8^+^ T-cell subsets: central memory T (TCM), effector memory T (TEM), and terminal effector T (TTE) cells **D-F.** Dot plot showing differential expression levels of genes in the B cell subtypes **(D)**, CD4T cell subtypes **(E)**, and CD8T cell subtypes **(F)**. Figure descriptions are the same as Fig. 2B-D. **G.** The plasma levels of CRIP1, IFITM1, TNF-α, CRP, and ESR in healthy controls and patients with periodontitis were measured using ELISA. P-values were calculated using the Wilcoxon rank-sum test to compare the two groups **H.** Relationship between CRIP1 and TNF-α, CRP, and ESR measurements. The Pearson correlation coefficient and p-values were calculated from the correlation test **I.** Receiver operating characteristic (ROC) curves of CRIP1 and IFITM1 ROC curve analysis showed a clear distinction between the healthy and periodontitis groups

Next, we investigated the transcriptomic changes in adaptive immune cells. The B, CD4T, and CD8T cell groups from Fig. 1 C were extracted and separated into more specific subtypes. B cells were separated into non-switched memory B cells (non-switched MB), switched memory B cells (switched MB), exhausted B cells, and plasmablasts (Fig. 3 A). CD4^+^ T cells were divided into four subtypes: T helper 1 (Th1), T helper 17 (Th17), follicular helper T (Tfh), and regulatory T (Treg) cells (Fig. 3B). CD8^+^ T cells were classified into three subtypes: central memory T (TCM), effector memory T (TEM), and terminal effector T (TTE) cells (Fig. 3 C). The markers for each compartment are described in Additional file 6: Fig. S3B‒D.

The genes that showed altered expression levels in the pre-treatment samples compared to those in both the healthy and post-treatment samples are shown in Fig. 3D‒F. The GO terms of these genes are described in Additional files 9‒11: Fig. S6‒S8. The CD4^+^ and CD8^+^ T cell subsets showed decreased cell migration and motility in pre-treatment patients. However, there were no notable changes in the expression of exhausted B cells, plasmablasts, and TEM cells. The lack of differences in TEM cells between the groups was expected since TEM cells were previously reported to be a part of the acute (but not chronic) immune response [24]. This suggests that these nonresponsive cells are not affected by the periodontal bacteria-induced systemic inflammation and are not the main drivers of chronic inflammation.

### Common DEGs in immune cells are responsive to systemic chronic inflammation

Across all immune cells, we found common DEGs, such as *CRIP1*, macrophage migration inhibitory factor (*MIF*), interferon-induced transmembrane protein 1 (*IFITM1*), and ribosomal protein S17 (*RPS17*). DEGs that exhibited contrasting expression patterns in the pre-treatment group compared to those in the healthy and post-treatment groups and their GO terms in the remaining cell types that were not divided into subtypes are shown in Additional files 12, 13: Fig. S9, S10. Notably, in more than half of the cells, all four genes were highly expressed only in pre-treatment patients (Additional File 14: Table S4). The relative expression of these four genes in the healthy group was visualized along with their localization according to clinical inflammatory and periodontal measurements in Additional file 15: Fig. S11. Across all cell types where differential expression was observed, the expression of the four genes increased. *CRIP1* was specifically upregulated in the B cell lineage, and *IFITM1* was upregulated in the T cell lineage. However, they were all reduced following therapeutic intervention, thus demonstrating a relationship between clinical variables and the expression patterns of these genes.

Of the four genes, *CRIP1* and *IFITM1* were overexpressed in approximately 97% and 71% of the total immune cells, respectively (Additional file 14: Table S4). An ELISA was conducted on the plasma of the healthy and periodontitis groups to measure changes in CRIP1 and IFITM1 protein concentrations, and compared with those of the inflammatory markers TNF-α, CRP, and ESR. Although there was a similar concentration of the three inflammatory indicators between the healthy and periodontitis groups, we found a difference in CRIP1 and IFITM1 levels (Fig. 3G). In addition, CRIP1 levels positively correlated with TNF-α levels (Fig. 3 H, Additional file 16: Fig. S12). The ROC curves showed that the area under the ROC curve (AUC) for CRIP1 and IFITM1 in the healthy and periodontitis groups was 0.94 and 0.66, respectively (Fig. 3I, both p-values < 0.001). Thus, these results indicate that the levels of these four genes are sensitive to the systemic immune response and that plasma CRIP1 could be a marker for the systemic inflammation caused by periodontitis.

### Cell‒cell communication in immune cells of patients with chronic periodontitis

To investigate the intercellular communication between immune cells, which could induce or suppress inflammatory signals, we inferred the cell**‒**cell interactions within each group. We then compared the signal length of the interactions between the groups (Additional file 17: Fig. S13) and found several chronic inflammation-specific crosstalk proteins: B and T lymphocyte attenuator (BTLA), CCL, CD99, SEMA4, RESISTIN, interferon-γ (IFNG), THBS, and CD48.

### Changes in the BTLA and IFNG pathways by periodontal infection are reversible with treatment

**Fig. 4:**
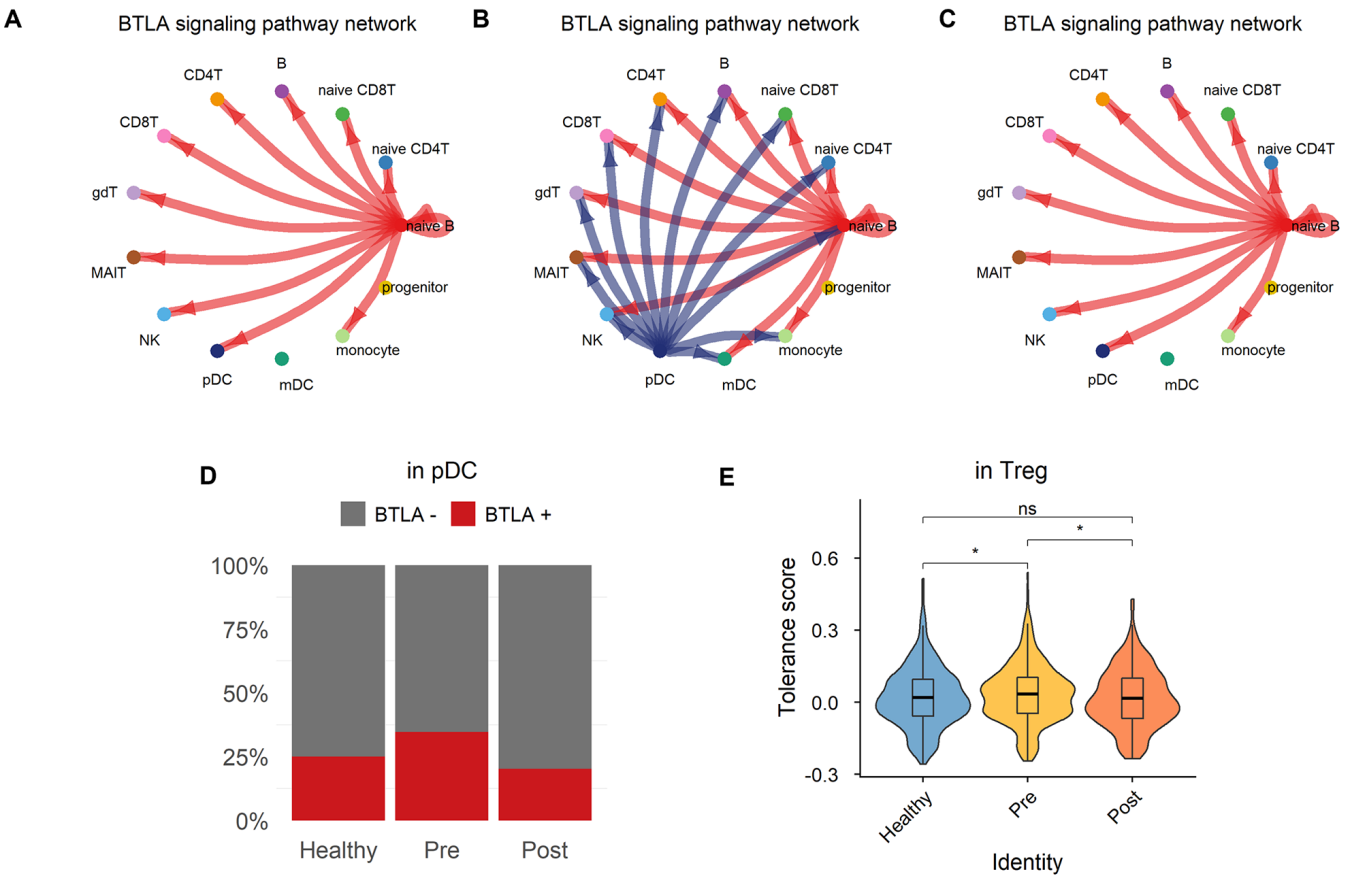
**BTLA signaling pathway and proportion of BTLA**^**+**^ **pDCs** **A–C.** Circle plot of BTLA signaling in healthy controls **(A)**, pre-treatment **(B)**, and post-treatment **(C)** groups. The direction of the arrow indicates the signal sender and receiver and the color of the arrow is the same as that of the sender cell. The edge width corresponds to the strength of the ligand-receptor pairs **D.** Proportion of BTLA^+^ pDCs in healthy, pre- and post-treatment groups **E.** Violin plot for the tolerance score of Tregs. Tolerance was calculated using the gene set from GO:0002645 (termed ‘positive regulation of tolerance induction’). Asterisks denote the significance of the differences between the groups calculated using the Wilcoxon rank-sum test. p ≤ 0.05 (*), p ≤ 0.01 (**), p ≤ 0.001 (***), p ≤ 0.0001 (****), p ≥ 0.05 (ns)

The BTLA-mediated pathway was significantly upregulated in pre-treatment patients. The BTLA pathway consists of BTLA‒TNFRSF14 as a ligand‒receptor pair in every group, and naïve B cells predominantly acted as the signal senders (Fig. 4 A‒C). However, unlike the other groups, pDCs also exerted a sending function. In addition, the proportion of pDCs expressing BTLA was higher in the pre-treatment group (Fig. 4D). Based on a study where BTLA‒TNFRSF14 crosstalk promoted Foxp3 expression in T cells via upregulation of CD5, we examined the proportional change in CD5^+^-expressing T cells and FOXP3^+^ Tregs. As a result, the ratio of CD5^+^ T cells and FOXP3^+^ Tregs increased in the periodontitis pre-treatment group (Additional file 18: Fig. S14). Moreover, in Treg cells, there was a significantly higher propensity for immune tolerance in the pre-treatment group (Fig. 4E). This is consistent with a previous study showing that BTLA in DCs modulates the immunotolerance of T cells [25]. After non-surgical treatment, the BTLA^+^ pDC ratio and the involvement of pDCs in BTLA interactions, and the tolerance of Tregs, were similar to those of the healthy group.

**Fig. 5:**
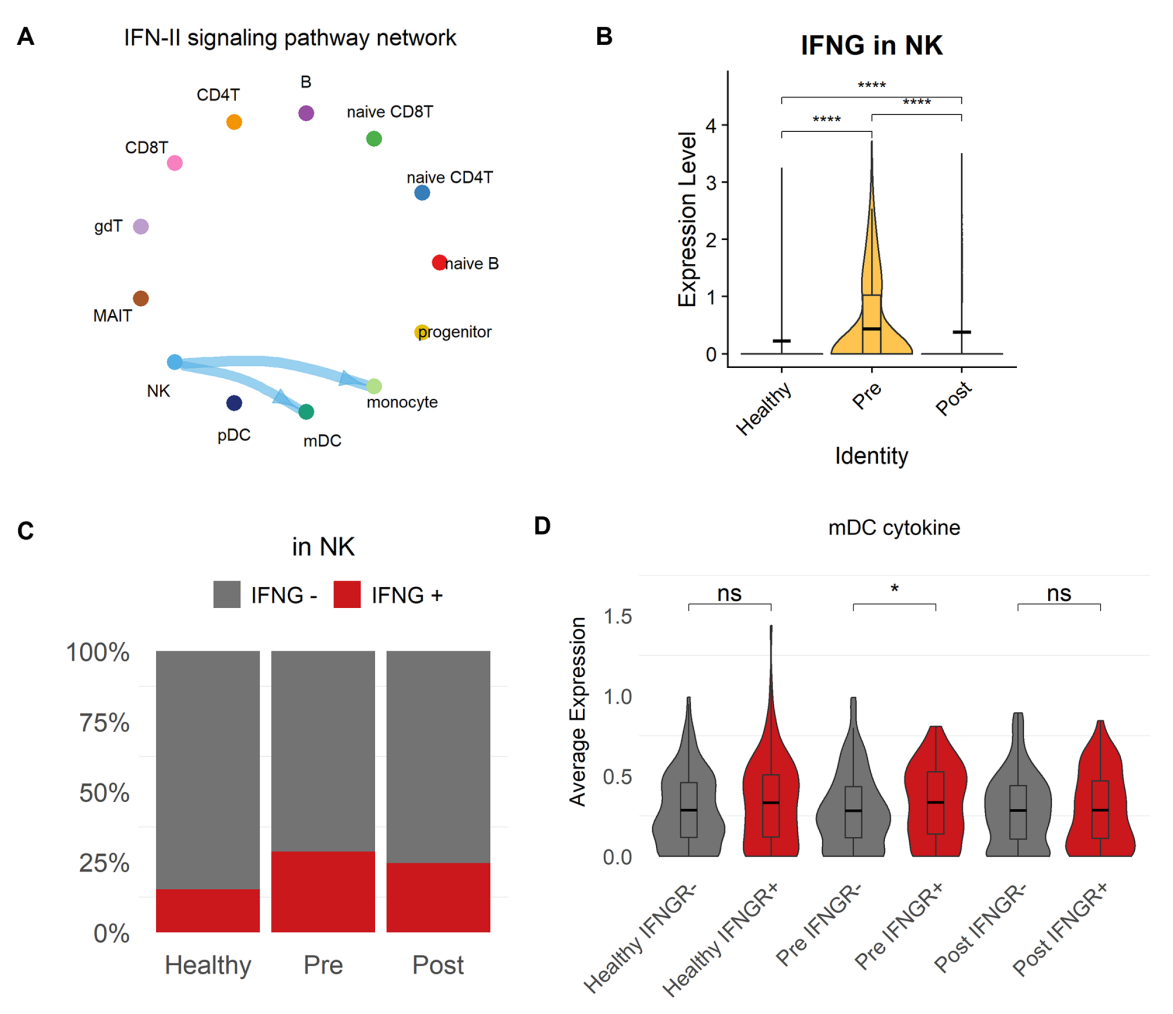
**Circle plot and expression level of IFNG signaling pathway** **A.** Circle plot of IFNG signaling in the pre-treatment group, similar to Fig. 4 A‒C. The edge width corresponds to the strength of the ligand-receptor pairs **B.** Violin plot of *IFNG* expression levels. The horizontal line indicates the average in each group, and the asterisks denote the significance of the difference between groups calculated using the Wilcoxon rank-sum test. p ≤ 0.05 (*), p ≤ 0.01 (**), p ≤ 0.001 (***), p ≤ 0.0001 (****), p ≥ 0.05 (ns) **C.** Proportion of IFNG^+^ NK cells in the healthy, pre- and post-treatment groups **D.** Average expression of cytokines including *IL1*, *IL12*, *IL23*, and *TNF-α* in dendritic cells by condition and IFNG receptor expression. The horizontal line indicates the average in each group, and asterisks denote the significance of the difference between groups calculated using the Wilcoxon rank-sum test. p ≤ 0.05 (*), p ≤ 0.01 (**), p ≤ 0.001 (***), p ≤ 0.0001 (****), p ≥ 0.05 (ns)

Conversely, IFNG-mediated interactions only appeared in the periodontal inflammatory group (Fig. 5 A). IFNG secreted by NK cells binds to IFNG receptors (IFNGR) on mDCs and monocytes. The expression level of IFNG and the ratio of IFNG^+^ NK cells were the highest in the pre-treatment group (Fig. 5B, C). In addition, there were significant increases in pro-inflammatory cytokines released from DCs only in the IFNGR^+^ mDCs of pre-treatment individuals (Fig. 5D) [26]. This suggests that NK cell-secreted IFNG stimulates mDCs to induce and maintain an inflammatory response in patients with periodontitis but can be ameliorated by non-surgical therapy [27, 28].

### Persistent RESISTIN pathway activation despite periodontitis treatment

**Fig. 6:**
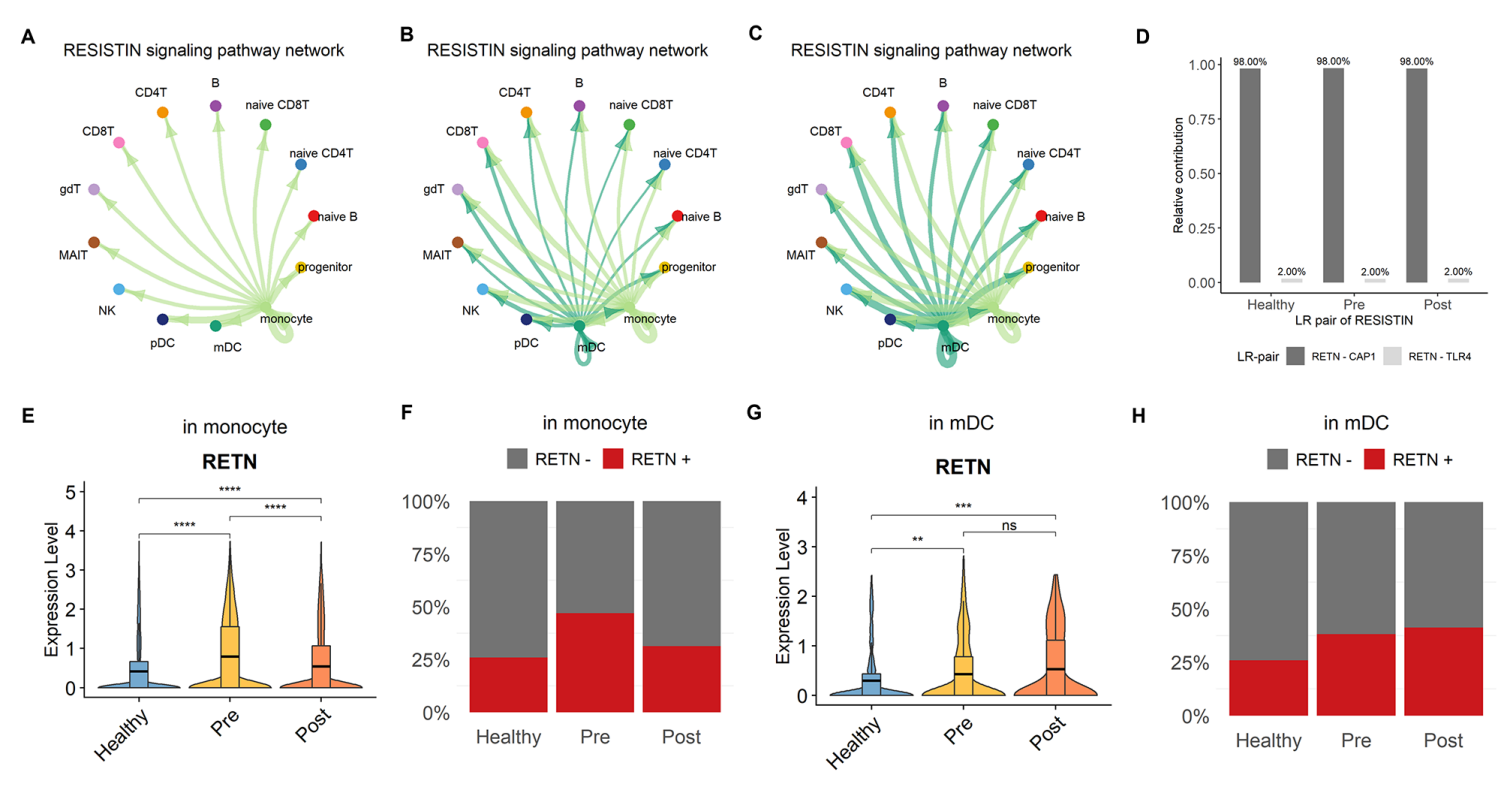
**Circle plot and expression level of RESISTIN signaling pathway** **A-C.** Circle plot of RESISTIN signaling in the healthy **(A)**, pre-treatment **(B)**, and post-treatment **(C)** groups, similar to Fig. 4 A‒C. The edge width corresponds to the strength of the ligand‒receptor pairs **D.** Relative contribution of ligand-receptor pairs for RESISTIN signaling pathway **E.** Violin plot of *RETN* expression levels in monocytes. The horizontal line indicates the average in each group, and the asterisks denote the significance of the difference between groups calculated using the Wilcoxon rank-sum test. p ≤ 0.05 (*), p ≤ 0.01 (**), p ≤ 0.001 (***), p ≤ 0.0001 (****), p ≥ 0.05 (ns) **F.** Proportion of RETN^+^ monocytes in the healthy, pre- and post-treatment groups **G.** Violin plot of *RETN* expression levels in mDCs. The horizontal line indicates the average in each group, and the asterisks denote the significance of the difference between groups calculated using the Wilcoxon rank-sum test. p ≤ 0.05 (*), p ≤ 0.01 (**), p ≤ 0.001 (***), p ≤ 0.0001 (****), p ≥ 0.05 (ns) **H.** Proportion of RETN^+^ mDCs in the healthy, pre- and post-treatment groups

The RESISTIN pathway was observed in all groups, with the weakest signal intensity in healthy subjects and increased signal intensity in periodontitis patients (Fig. 6 A‒C). In all groups, monocytes interact with all cell types as the signal sender, but in the disease state, mDCs are also able to initiate signal transduction. The RESISTIN pathway consists of interactions with CAP1 and TLR4 receptors, and there was no compositional difference in the ligand-receptor pairs between the groups (Fig. 6D). Examining the expression pattern of the resistin-coding gene *RETN* in signal sender cells, RETN was most highly expressed in monocytes of the periodontitis group, but levels were significantly decreased after treatment, although it was not completely rescued (Fig. 6E). In addition, the proportion of RETN^+^ monocytes in the periodontitis group was twice that of the healthy controls, but decreased to levels similar to the healthy group after treatment (Fig. 6 F). In contrast, in mDCs, the expression level of RETN and the proportion of RETN^+^ cells were only slightly increased (Fig. 6G, H). Therefore, it is noteworthy that, unlike the preceding signaling pathways, treatment intervention did not rescue the changes in RESISTIN pathway activity due to periodontitis. In conclusion, although there was partial rescue in some cells that activated the RESISTIN signaling pathway, mDCs involved in periodontitis were not affected by non-surgical treatment and continued to activate RESISTIN signaling.

In addition, there are other therapy-responsive and nonresponsive intercellular pathways. The CCL and SEMA4 signals, which were elevated in pretreatment patients, were weakened after periodontal treatment, while CD99, THBS, and CD48 signals were maintained. The ligand-receptor pair and cellular networks of these pathways are illustrated in Additional file 19: Fig. S15.

## Discussion

Periodontitis is caused by periodontal dysbiosis of the microbiota, which promotes loss of gingival tissue and bone destruction [29]. The risk of periodontitis affects not only the local site, but also induces a systemic immune response. For example, periodontitis-induced chronic inflammation can disturb typical immunological mechanisms, including bystander activation, amplification of cytokines, epitope spreading, autoantigen complementarity, and activation or inhibition of receptors related to microorganism regulation [29]. Consequently, pathogenesis models leading to extra-oral diseases, such as cardiovascular disease, rheumatoid arthritis, adverse pregnancy outcomes, and respiratory disease, have been widely investigated [30]. However, beyond the pathophysiology of periodontitis, the systemic immune response to periodontal treatment remains unclear. To expand our knowledge, this study shows the periodontal therapy-induced process and identifies genes that are differentially altered according to the disease condition. Moreover, we focused on the intercellular signals between immune cells and further uncovered the interactions that did not respond to periodontal treatment.

In this study, we examined the transcriptional changes and related functions in different immune cells. Each immune cell had pro-inflammatory cytokines that were elevated only in pre-treated periodontitis patients. For instance, CCL3L1 (in NK cells), which enhances the inflammatory response and increases the risk of autoimmune disease [31], S100A12 (in mDCs), which is a valuable serum inflammatory marker [32], TNFRSF1B (in mDCs), which regulates TNF-α levels [33], and CCR7 (in Th17 cells), which promotes joint inflammation [34], were found to be highly expressed in patients with periodontitis. Unexpectedly, we observed that only a few pro-inflammatory cytokines were altered in periodontitis. In addition, as shown by the ELISA results, the prevalent inflammatory markers only showed slightly increased levels in periodontitis. Altogether, our results indicate that periodontitis induces mild systemic inflammation, consistent with previous studies [7, 35].

Remarkably, the expression of the *CRIP1* gene was differentially increased in almost every cell type in the periodontitis samples and the concentration of plasma CRIP1 protein also increased in the periodontitis group. The functional role of CRIP1 has been reported in a previous study, where transgenic mice overexpressing *Crip1* produced high concentrations of IL-6 and IL-10 after LPS treatment [36]. In addition, its role has been studied in diverse cancer types, suggesting that *CRIP1* might be a risk factor for chronic inflammation and systemically affects numerous tissues and organs [37–40]. Moreover, CRIP1 levels reflect periodontitis-induced inflammation more sensitively than typical indicators. Taken together, the upregulation of *CRIP1* may be a predictive marker for systemic inflammation induced by periodontitis.

In the intercellular network, BTLA and IFNG signals are strongly activated in the circulatory system during periodontitis. The BTLA‒TNFRSF14 interaction has been reported to regulate T-cell responses and increase disease susceptibility. Here, we confirmed that pDCs formed distinct signaling networks, and the immunological tolerance of T cells was enhanced in periodontal disease [41]. It is speculated that this is because BTLA-expressing DCs induce and modulate subsequent immune responses of Treg cells by promoting Foxp3 and CD5 expression [25]. Additionally, mDCs and monocytes are involved in IFNG network. In particular, mDCs expressing IFNGR secrete high levels of inflammatory cytokines, which may lead to abnormal localization or activation of the immune response [28]. Similarly, the CCL pathway, which is known to augment the production of IFNG from the CCL5‒CCR1 interaction, was strongly upregulated in periodontitis patients, which supports the higher activity of INFG [42]. From these results, we can infer that patients with periodontitis exhibit antigen-specific tolerance and an imbalanced immune response. However, these intercellular networks can be resolved using nonsurgical periodontal treatment.

In contrast, the RESISTIN pathway was consistently active regardless of non-surgical treatment. Unlike rodent adipocytes, human RESISTIN is predominantly expressed in macrophages and is produced in response to inflammatory stimuli [43–46]. As in previous research, most RESISTIN transcripts were secreted by monocytes from healthy donors and patients with periodontitis. However, interaction with mDCs appeared in patients with periodontitis and was sustained even after periodontal therapy. In addition, RESISTIN binds primarily to the adenylyl cyclase-associated protein 1 (CAP1) receptor, which is known to upregulate circulating AMP (cAMP) concentrations, protein kinase A (PKA) activity, and NF-κB-associated transcription of inflammatory cytokines [47]. Elevated RESISTIN levels have also been demonstrated in various inflammatory diseases, such as rheumatoid arthritis (RA), inflammatory bowel disease (IBD), type II diabetes, and sepsis. [48–50]. Thus, RESISTIN signaling may systemically increase susceptibility to diverse diseases. Moreover, the THBS network, whose ligand is highly expressed after LPS stimulation, increases the risk of inflammation [51, 52], and the CD48 network, which is known to activate NK cells and contribute to diverse autoimmune diseases, is maintained even after periodontal therapy [53, 54]. All this evidence indicates that typical periodontal treatment cannot be the sole strategy for complete immune recovery and necessitates consideration of other therapeutic approaches.

Nevertheless, this study had some limitations. First, there is a gap between the ages of healthy individuals and patients with periodontitis. Age is one of the major factors that enhance the prevalence of periodontitis and chronic inflammation [55–57]. Although all subjects in this study were middle-aged, there was a 6-year difference between the groups on average. This could indicate that the patients had higher exposure to a known risk factor for chronic inflammation; thus, further research under strict control of external factors is required. Next, cryopreserved PBMCs were used to generate RNA sequence profiles. Despite the general use of frozen samples in scRNA-seq, the identification of immune cell subpopulations that have not been fully characterized may be challenging, considering the comparatively lower detection of UMIs in frozen PBMCs than in fresh PBMCs [58, 59]. Therefore, fresh samples can assist with producing more precise results.

## Conclusion

In summary, our current study reports transcriptome changes in periodontitis at single-cell resolution. In this study, we revealed the systemic immunological effects of periodontitis and identified periodontitis-specific predictors of inflammation. In addition, based on the immune pathways that were responsive to therapy, we found nonresponsive pathways to treatment that can increase the risk of comorbidity. Therefore, we suggest target pathways that can resolve chronic systemic inflammation but these therapeutics targets will require experimental validation.

## Electronic supplementary material

Below is the link to the electronic supplementary material.

Supplementary Material 1

Supplementary Material 2

## Data Availability

The datasets generated during this study are available at GEO (https://www.ncbi.nlm.nih.gov/geo/) under accession number GSE174609.

## Abbreviations

AUC: area under the ROC curve
BTLA: B and T lymphocyte attenuator
CAL: clinical attachment level
CRIP1: cysteine-rich protein 1
CRP: C-reactive protein
DC: dendritic cell
DEG: differentially expressed gene
ELISA: Enzyme-linked immunosorbent assay
ESR: erythrocyte sedimentation rate
GI: gingival index
GO: gene ontology
IFITM1: interferon-induced transmembrane protein 1
IFNG: interferon-γ
IFNGR: IFNG receptor
LPS: lipopolysaccharide
mDC: myeloid dendritic cell
MIF: macrophage migration inhibitory factor
non-switched MB: non-switched memory B cells
PBMCs: peripheral blood mononuclear cells
pDC: plasmacytoid dendritic cell
PI: plague index
PPD: probing pocket depth
ROC curve: receiver operating characteristic curve
RPS17: ribosomal protein S17
scRNA-seq: single-cell RNA sequencing
switched MB: switched memory B cells
TCM: central memory T
TEM: effector memory T
Tfh: follicular helper T
Th1: T helper 1
Th17: T helper 17
Treg: regulatory T
TTE: terminal effector T
