# Supplementary material for: Single-cell RNA sequencing reveals rebalancing of immunological response in patients with periodontitis after non-surgical periodontal therapy
Source: J Transl Med. 2022 Nov 3;20:504. doi: 10.1186/s12967-022-03702-2 (PMC9635198; doi:10.1186/s12967-022-03702-2)
Supplement: Supplementary file 1 — Supplementary Material 1 [file 12967_2022_3702_MOESM1_ESM.docx]

Additional file for

Single-cell RNA sequencing reveals rebalancing of immunological response in patients with periodontitis after non-surgical periodontal therapy

Hansong Lee¶, Ji-Young Joo¶, Dong Hyun Sohn, Junho Kang, Yeuni Yu, Hae Ryoun Park*, Yun Hak Kim*

Correspondence to: Hae Ryoun Park mail to:parkhr@pusan.ac.kr and

Yun Hak Kim mail to yunhak10510@pusan.ac.kr

**This PDF file includes:**

Additional file 1 to Additional file 19

Additional file 1

Table S1 Clinical information of the participants included in scRNA-seq.

|  | Total | healthy | PD(Pre-tx) | PD(Post-tx)* |
| --- | --- | --- | --- | --- |
| patient (n) | 8 | 4 | 4 | 4 |
| male | 4 | 1 | 3 | 3 |
| female | 4 | 3 | 1 | 1 |
| Age, y (mean±sd) |  | 44.25±2.22 | 50±10.56 | 50±10.56 |
| CRP (mg/L) |  | 0.00 | 2.00 ±1.93 | 1.60±1.42 |
| ESR (mm/hr) |  | 2.50±1.73 | 18.00±13.64 | 11.50±8.96 |
| all probing pocket depth (mm) |  | 1.84±0.11 | 3.59±1.27 | 3.11±1.23 |
| ≥4mm probing pocket depth (mm) |  |  | 5.91±1.43 | 5.22±1.26 |
| clinical attachment level (mm) |  | 2.09±0.08 | 4.58±1.27 | 3.94±1.40 |
| plague index |  | 16.53±8.34 | 43.34±13.83 | 35.31±18.53 |
| gingival index |  | 0 | 0.70±0.24 | 0.33±0.17 |

* Paired sampling with pre-treatment patients

Additional file 2

Table S2 Clinical information of the subjects included in measurement of CRIP1 levels by ELISA.

|  | Total | healthy | PD | p-value |
| --- | --- | --- | --- | --- |
| patient (n) | 30 | 18 | 12 |  |
| male | 19 | 13 | 6 |  |
| female | 11 | 5 | 6 |  |
| CRP (mg/L) (mean±sd) |  | 0.89 ± 1.74 | 0.32 ± 0.42 | 0.63 |
| ESR (mm/hr) |  | 5.56 ± 4.34 | 6.92 ± 4.52 | 0.45 |

P-values were calculated using the Wilcoxon rank-sum test.

Additional file 3

Table S3 Clinical information of the subjects included in ELISA for IFITM1 measurement.

|  | Total | healthy | PD | p-value |
| --- | --- | --- | --- | --- |
| patient (n) | 40 | 18 | 22 |  |
| male | 26 | 13 | 13 |  |
| female | 14 | 5 | 9 |  |
| CRP (mg/L) (mean±sd) |  | 0.89 ± 1.74 | 0.94 ± 0.82 | 0.18 |
| ESR (mm/hr) |  | 5.56 ± 4.34 | 6.38 ± 7.28 | 0.93 |

P-values were calculated using the Wilcoxon rank-sum test. The healthy participants were identical to the individuals of the CRIP1 ELISA (Table S2).

Additional file 4

**
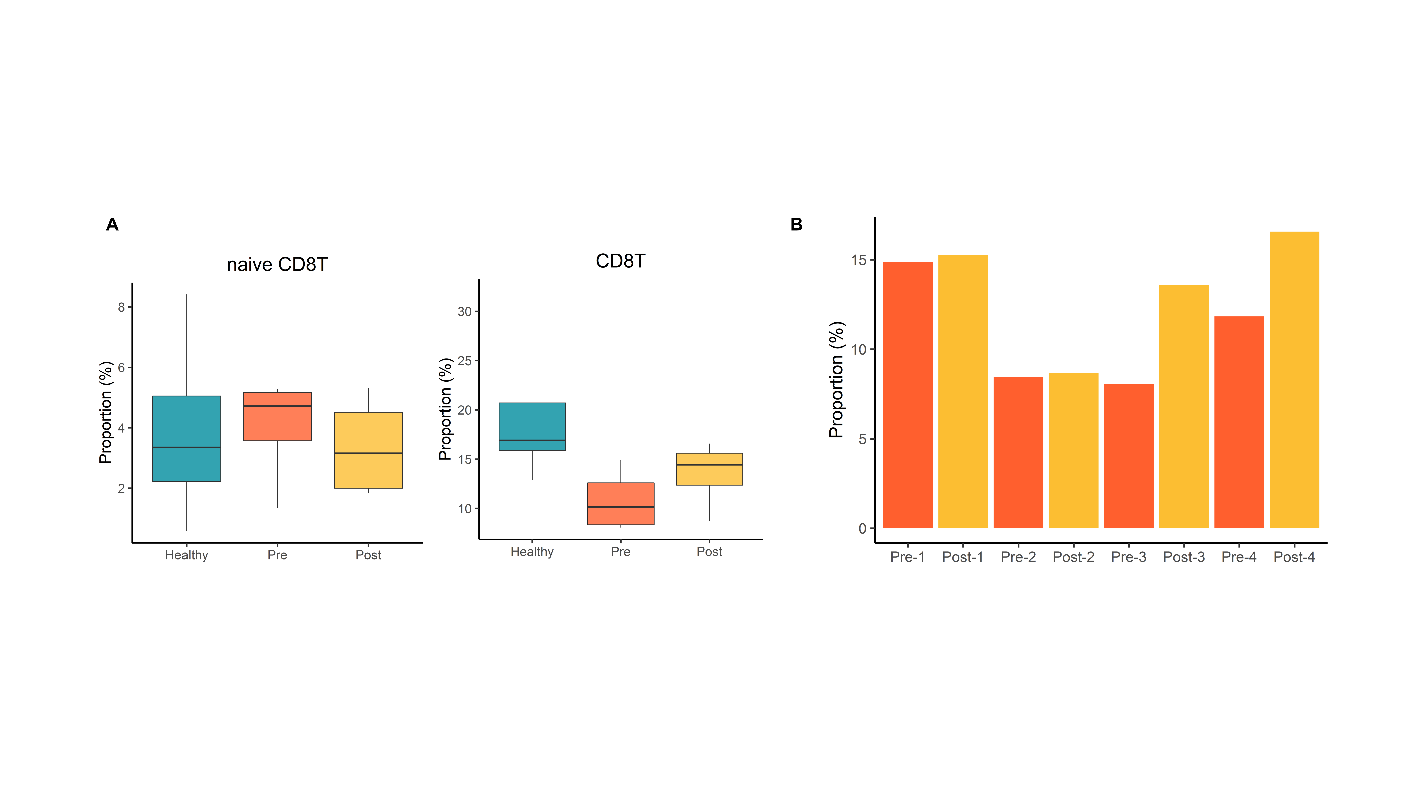
**

**Fig. S1 Proportion change in CD8^+^ T cells after periodontal therapy**

**A.** Box plot of the naïve CD8T and CD8^+^ T cell proportions for each group.

**B.** Bar graph of CD8^+^ T cell proportion showing an increasing trend after periodontal therapy for individual patients.

**Additional file 5**

**
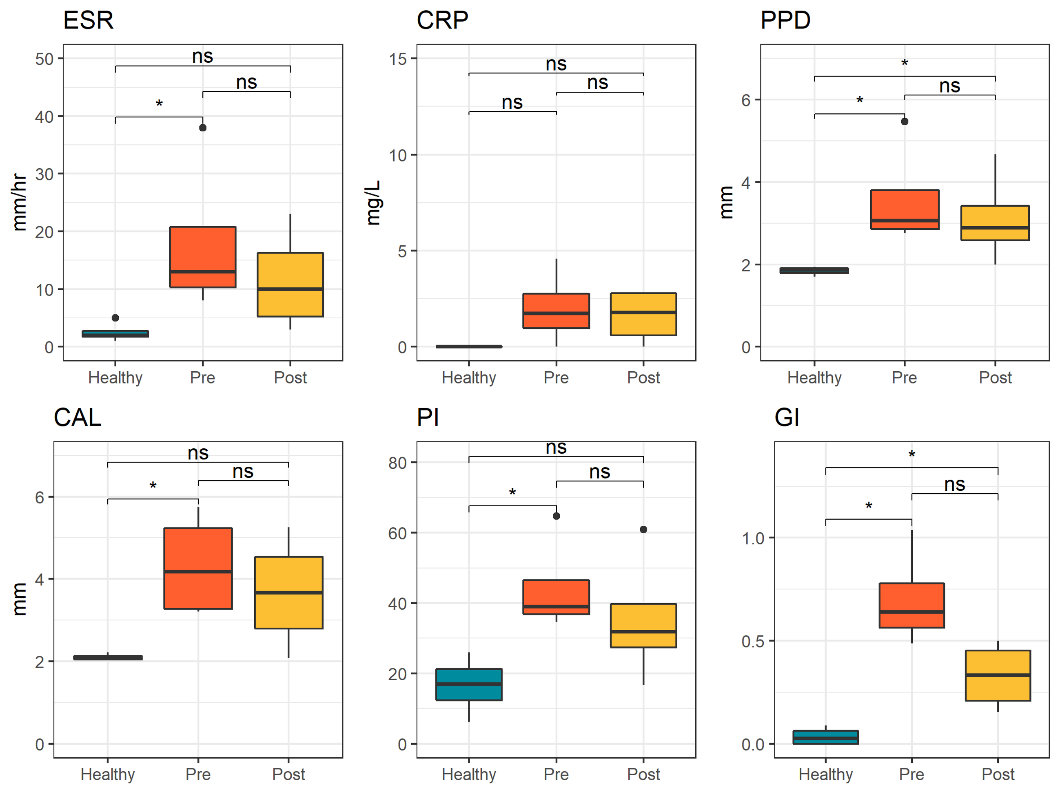
**

**Fig. S2 Boxplots of six clinical variables for healthy, pre-, and post-treatment groups**

The boxplots showing differences among the groups in erythrocyte sedimentation rate (ESR), C-reactive protein (CRP), probing pocket depth (PPD), clinical attachment level (CAL), plaque index (PI), and gingival index (GI). p ≤ 0.1 (•), p ≤ 0.05 (*), p ≤ 0.01 (**), p ≤ 0.001 (***), p ≤ 0.0001 (****), p ≥ 0.05 (ns); Wilcoxon rank-sum test.

Additional file 6


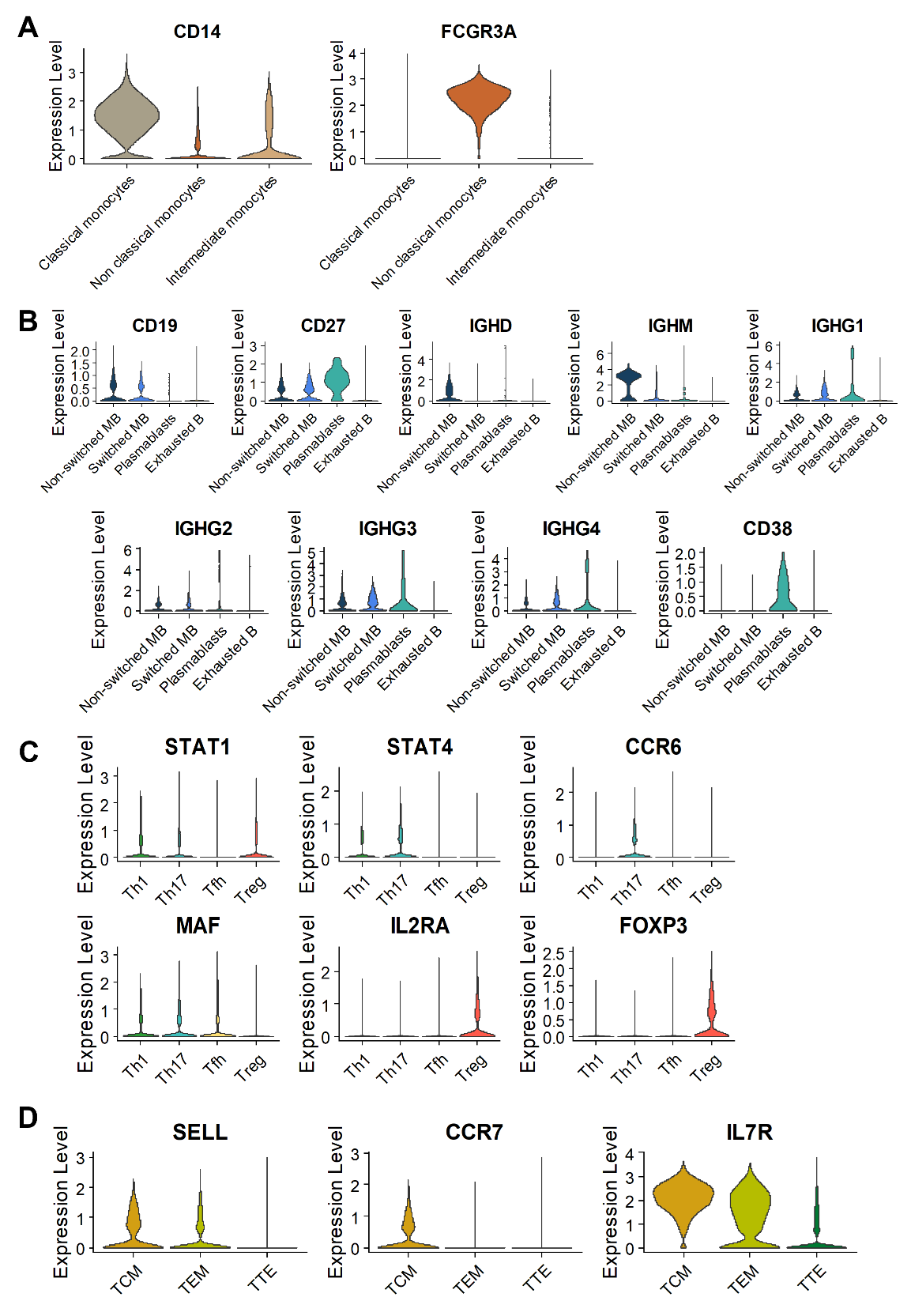


Fig. S3 Cellular markers for monocytes, B, CD4T, and CD8T cell subsets

A. Violin plot of the representative marker genes for the monocyte subtypes. Classical monocytes express CD14^++^FCGR3A(CD16)^−^, non-classical monocytes and intermediate monocytes express CD14^+^FCGR3A^++^ and CD14^++^FCGR3A^+^ respectively.

B. Violin plot of representative marker genes for the B cell subtypes. Non-switched MB and switched MB cells both express CD19 and CD27, but can be distinguished using IGHD and IGHM, which show low expression in switched MB cells and plasmablasts related to CD38.

C. Violin plot of the representative marker genes for the CD4T cell subtypes. STAT1 and STAT4 were used to identify Th1 cells, CCR6 for Th17 cells, MAF for Tfh cells, and IL2RA and FOXP3 for Treg cells.

D. Violin plot of the representative marker genes for the CD8T cell subtypes. The cells were grouped into $\mathbf{SELL}^{\mathbf{hi}}$/ $\mathbf{CCR7}^{\mathbf{hi}}$ central memory T (TCM), $\mathbf{SELL}^{\mathbf{lo}}$($\mathbf{CD62L}^{\mathbf{lo}}$)/ $\mathbf{CCR7}^{\mathbf{lo}}$effector memory T (TEM), and $\mathbf{SELL}^{\mathbf{lo}}$($\mathbf{CD62L}^{\mathbf{lo}}$)/ $\mathbf{CCR7}^{\mathbf{lo}}$/ $\mathbf{IL7R}^{\mathbf{lo}}$ terminal effector T (TTE) cells.

Additional file 7


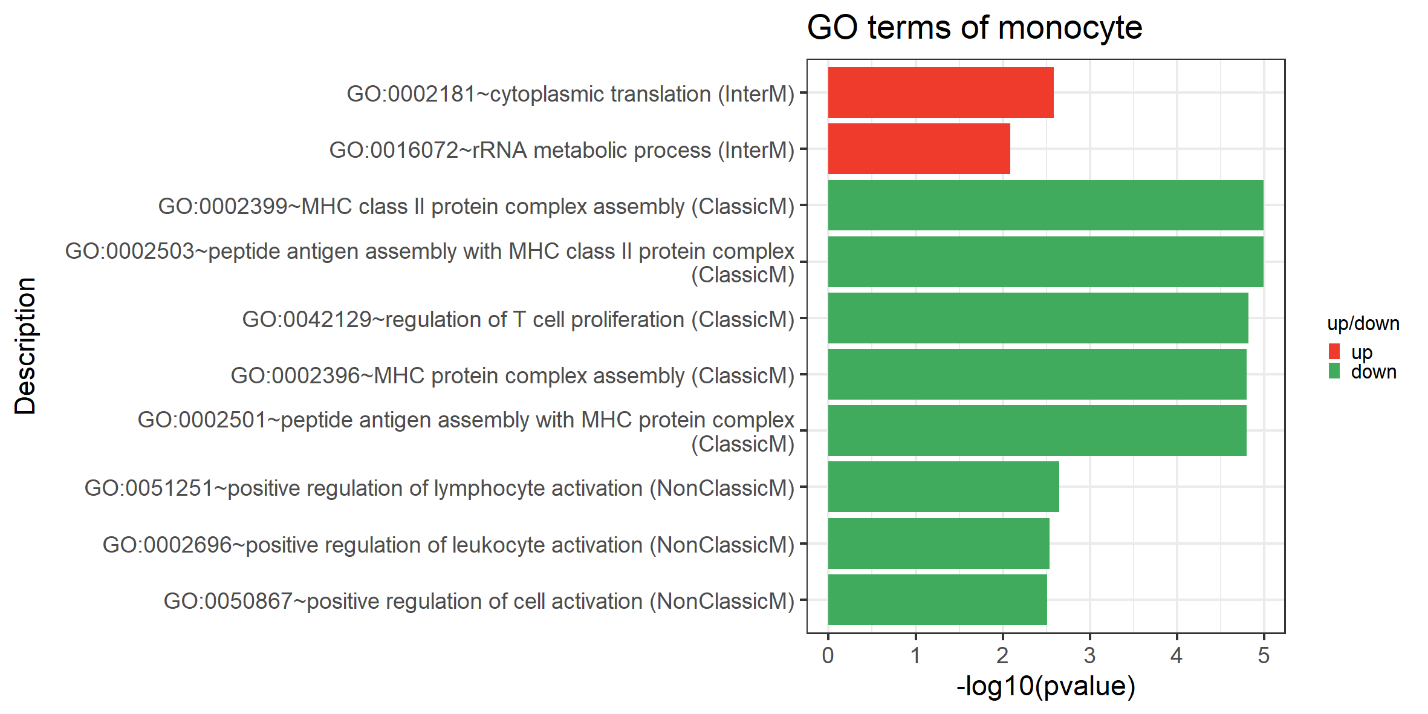


Fig. S4 GO terms for differentially expressed genes in monocyte subtypes detected in periodontitis.

Additional file 8


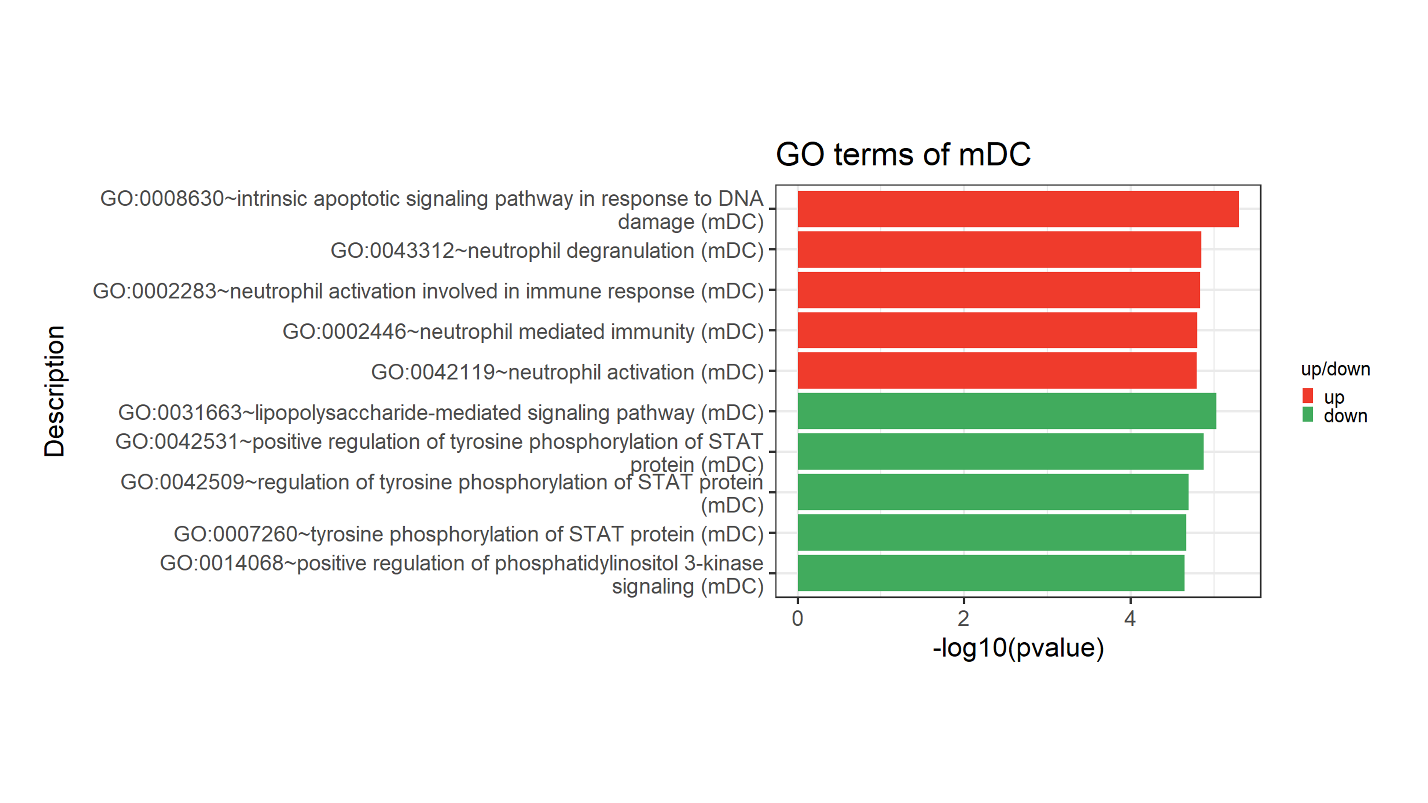


Fig. S5 GO terms for differentially expressed genes in mDCs detected in periodontitis.

Additional file 9


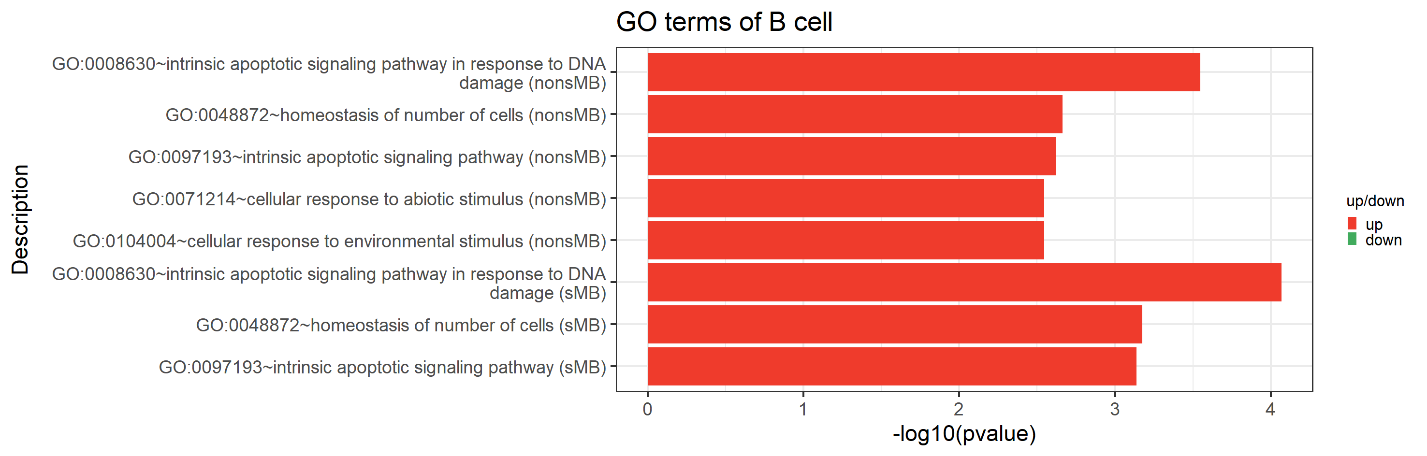
Fig. S6 GO terms for differentially expressed genes in B cell subgroups detected in periodontitis.

Additional file 10


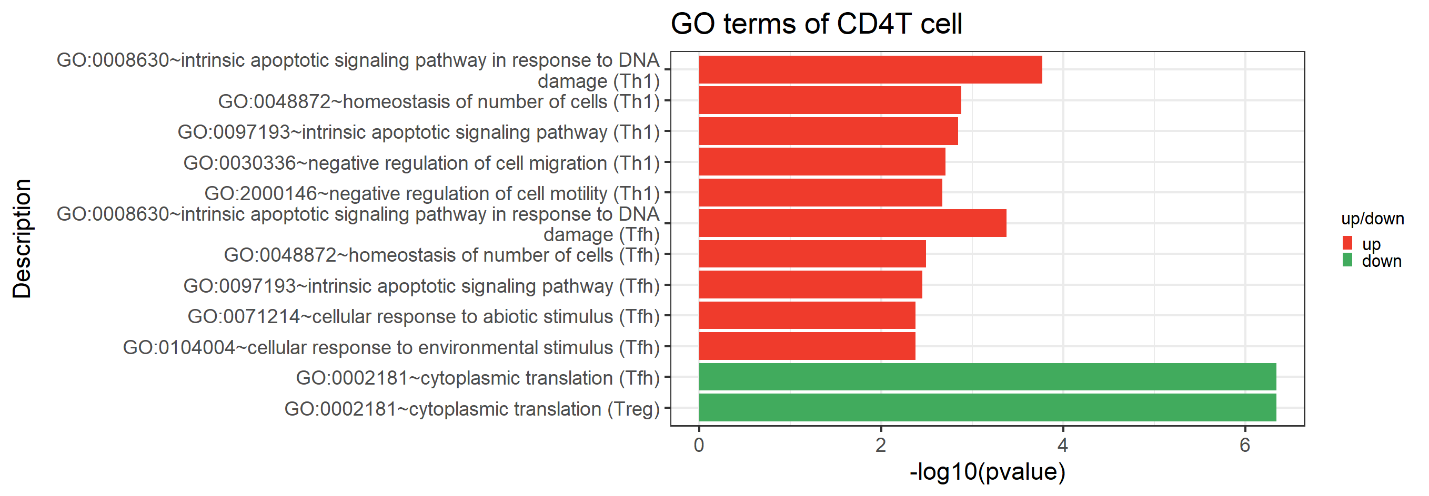


Fig. S7 GO terms for differentially expressed genes in CD4+ T cell subgroups detected in periodontitis.

Additional file 11
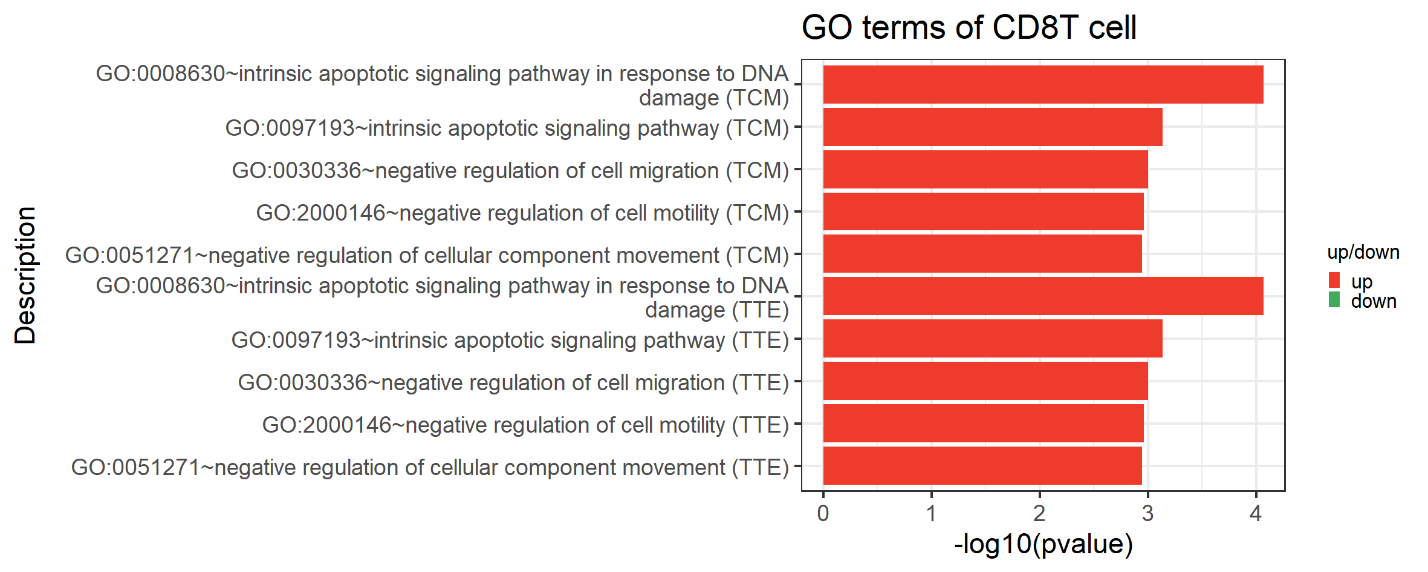
Fig. S8 GO terms for differentially expressed genes in CD8^+^ T cell subgroups detected in periodontitis.

Additional file 12


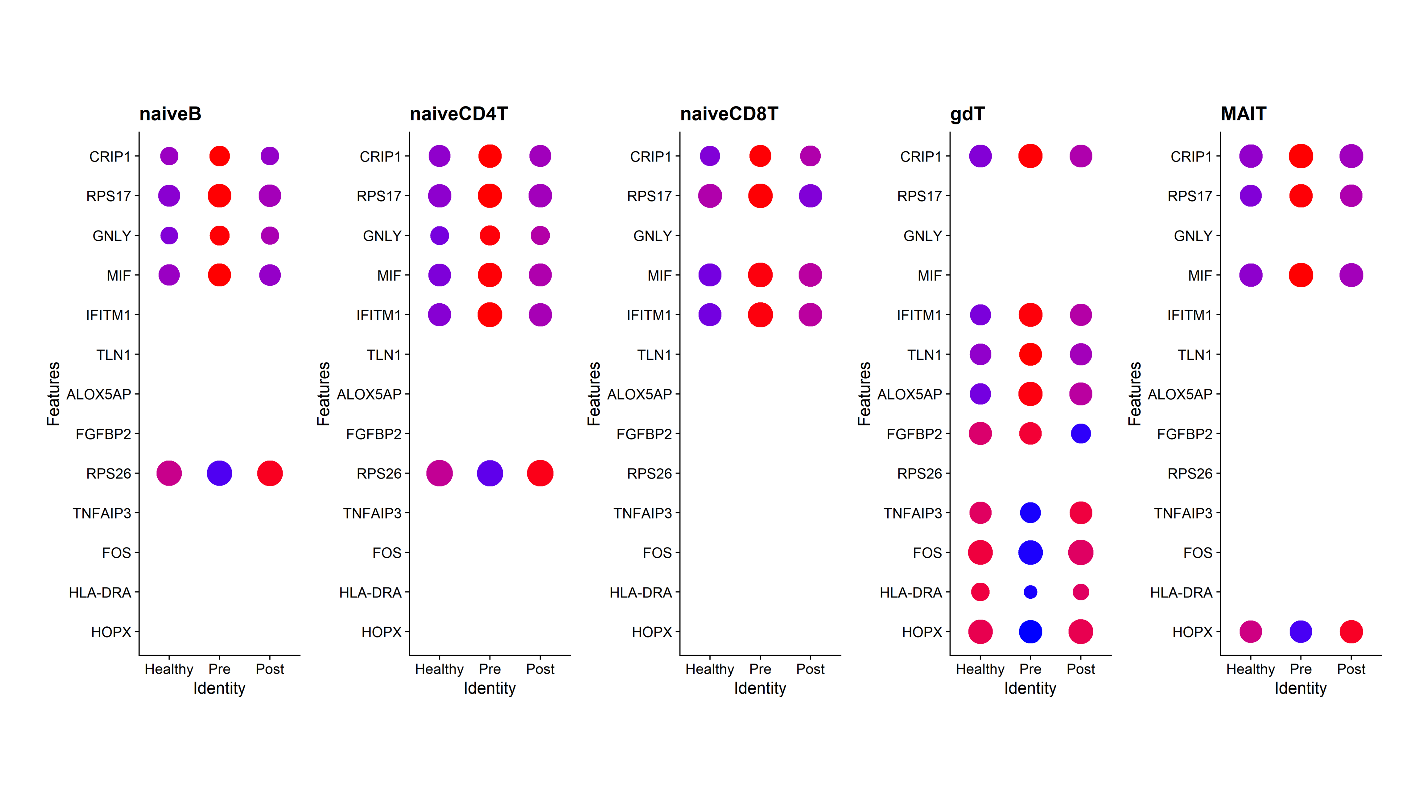


Fig. S9 Distinct gene expression patterns of naïve B, naïve CD4T, naïve CD8T, gdT, and MAIT cells.

Dot plot showing the differential expression levels genes for each cell type that was not divided into subtypes. Displayed genes showed similar expression in healthy and post-treatment groups but inverse expression patterns in the pre-treatment group. The color of the dots represent expression levels of the gene and dot size represents the fraction of cells expressing the gene in each specific cell type.

Additional file 13


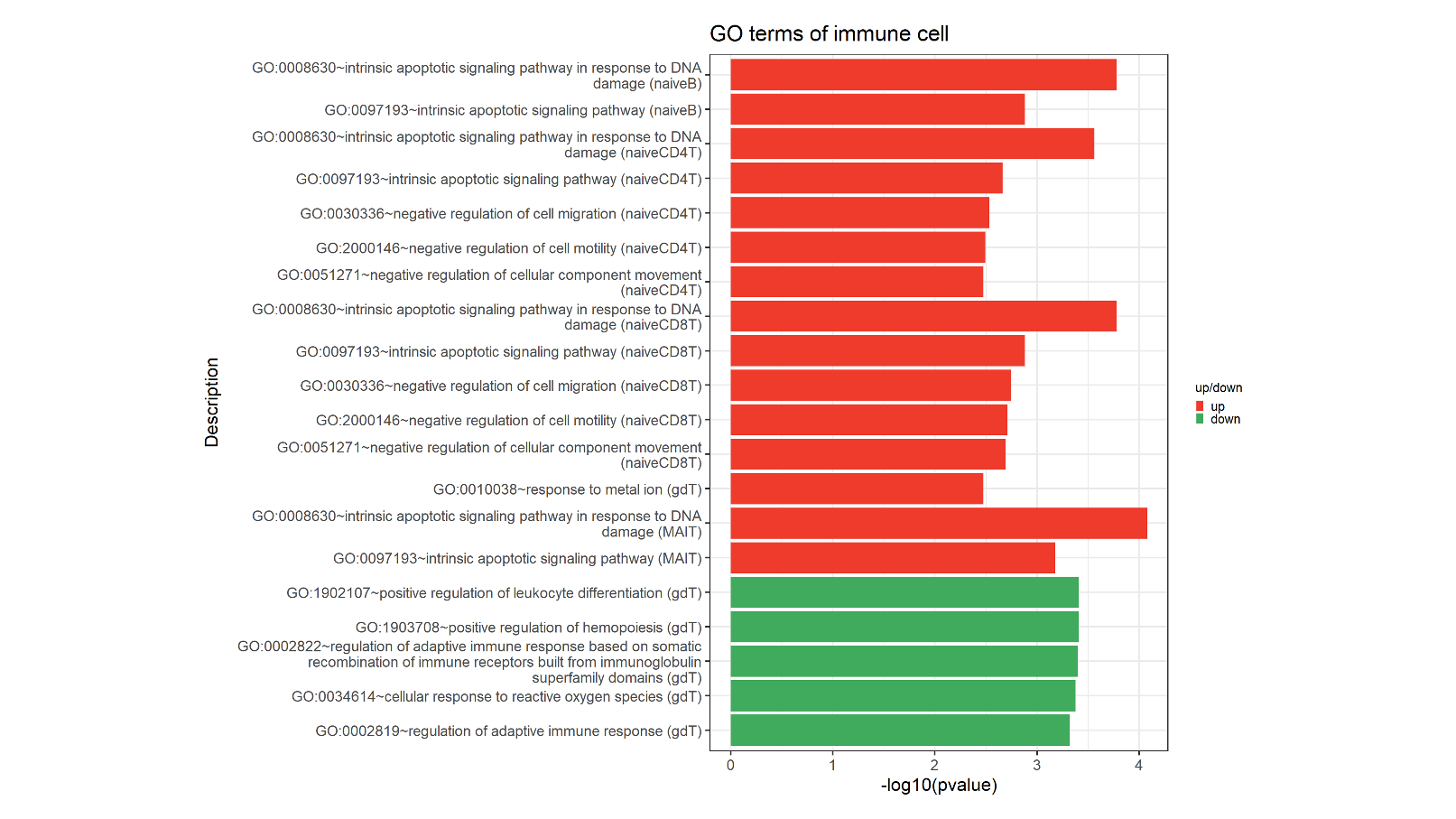


Fig. S10 GO terms for differentially expressed genes in other immune cells detected in periodontitis.

Additional file 14

Table S4 List of cell types expressing elevated level of CRIP1, IFITM1, MIF, and RPS17 in patients with periodontitis.

| Gene (number of detected cell types)  [percentage of the detected cells] | Differentially expressing cell type |
| --- | --- |
| CRIP1 (18) [96.56%] | naïve B, naïve CD4T, naïve CD8T, gdT, MAIT, NK, mDC, classical monocyte, non-classic monocyte, intermediate monocyte, Th1, Th17, Tfh, Treg, TCM, TTE, non-switched MB, switched MB |
| IFITM1 (11) [71.08%] | naïve CD4T, naïve CD8T, gdT, MAIT, NK, intermediate monocyte, Th1, Tfh, Treg, TCM, TTE |
| MIF (11) [63.89%] | naïve B, naïve CD4T, naïve CD8T, MAIT, mDC , Th1, Tfh, TCM, TTE, non-switched MB, switched MB |
| RPS17 (10) [53.69%] | naïve B, naïve CD4T, naïve CD8T, MAIT, mDC, intermediate monocyte, Th1, Tfh, non-switched MB, switched MB |

Additional file 15


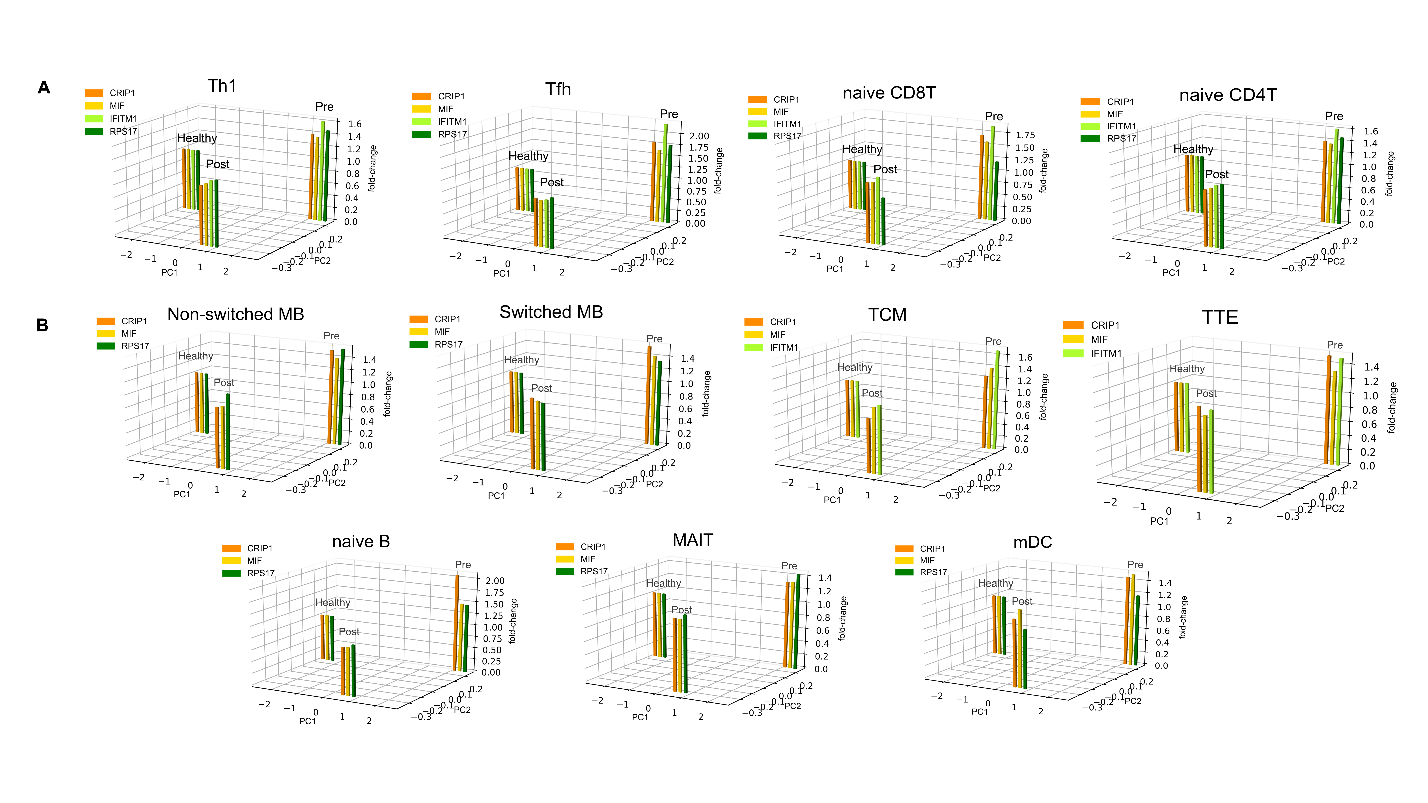


Fig. S11 The 3-dimensional plot for relative expression level of key genes according to pathological status.

A. The relative expression levels of 4 key genes on PCA axis using the average of 6 clinical variables (ESR, CRP, PPD, CAL, PI, and GI). The fold-change was calculated based on expression level of the periodontal group compared to that of the healthy group. All key genes are simultaneously detected in Th1, Tfh, naïve CD8T, and naïve CD4T cells.

B. The relative expression levels of key genes on PCA axis in each cell type where the three key genes are detected. Non-switched MB, switched MB, TCM, TTE, naïve B, MAIT, and mDC expression of the three key genes and PCA axis using the average of 6 clinical variables were generated the same as in A.

**Additional file 16**

**
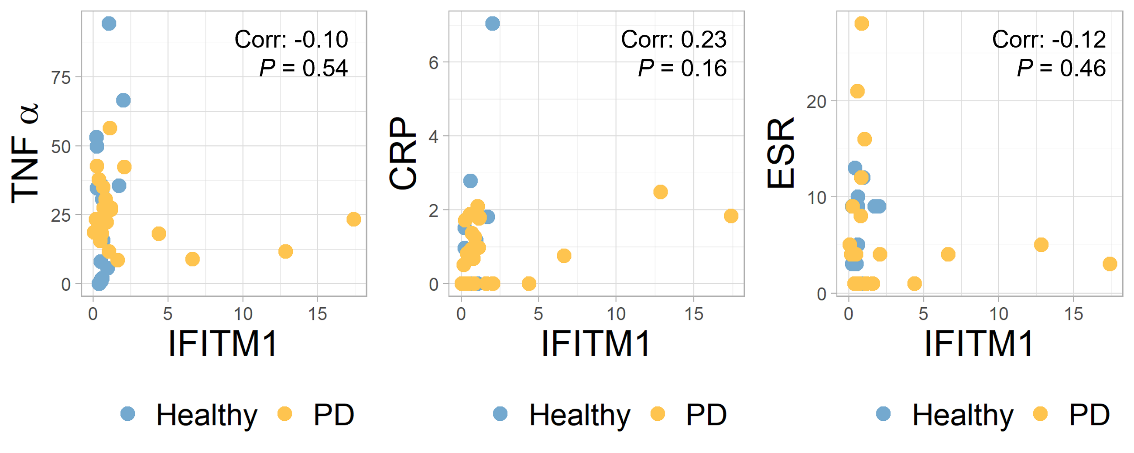
**

**Fig. S12 Correlation of** **IFITM1 expression with TNFα, CRP, and ESR levels**

The scatter plot displays the relationship between IFITM1 and TNF-α, CRP, and ESR levels measured by ELISA. The Pearson correlation coefficient and p-values were calculated from the correlation test. The groups are separated by color.

Additional file 17


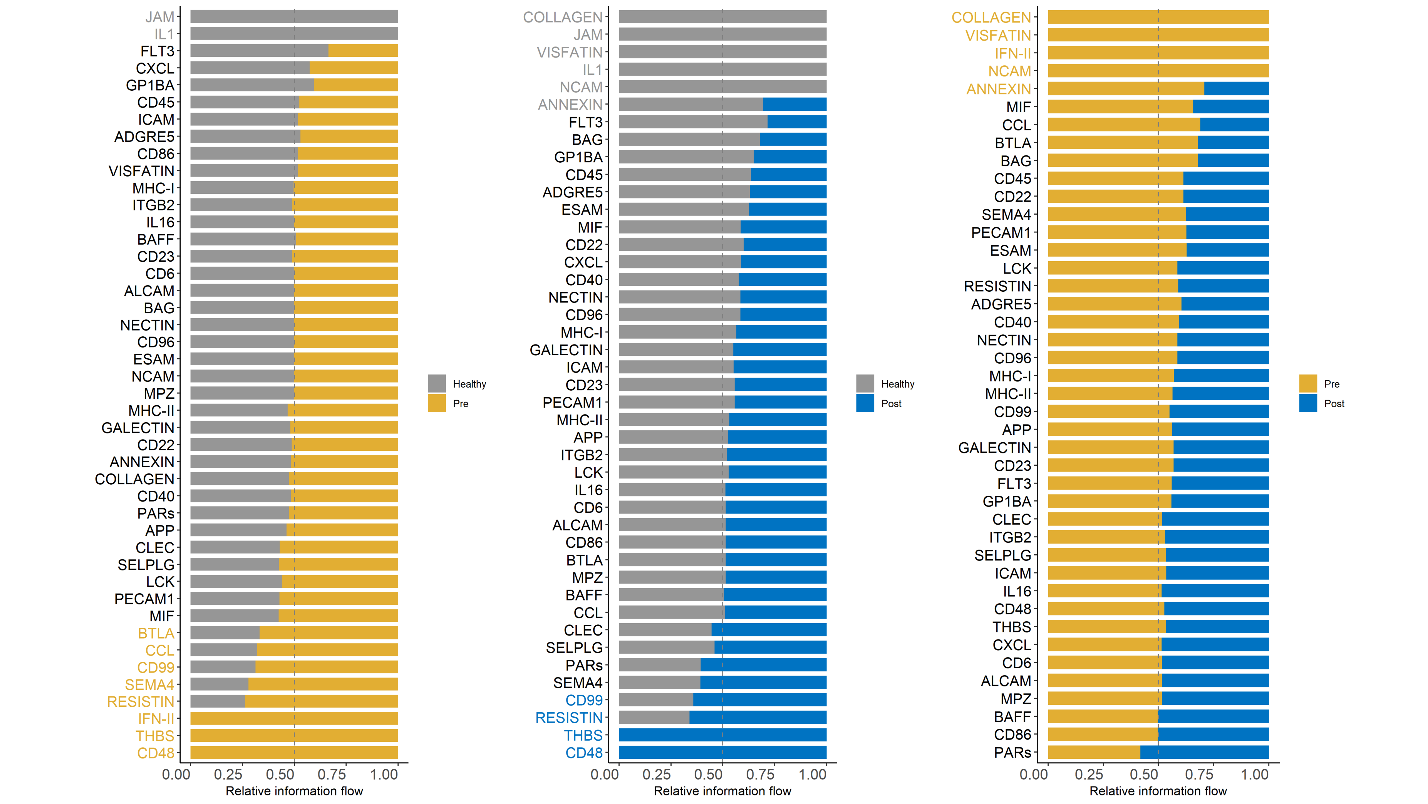


Fig. S13 Relative information flow of cell‒cell interactions.

The stacked bar graph of signaling pathways describes the relative contribution of each interaction based on the information flow, which is defined by the total weights among all pairs of cell groups in the inferred network. The colors of the pathway indicate the group in which the interaction is significantly detected, and pathways that are equally important in both datasets are indicated with black text.

Additional file 18


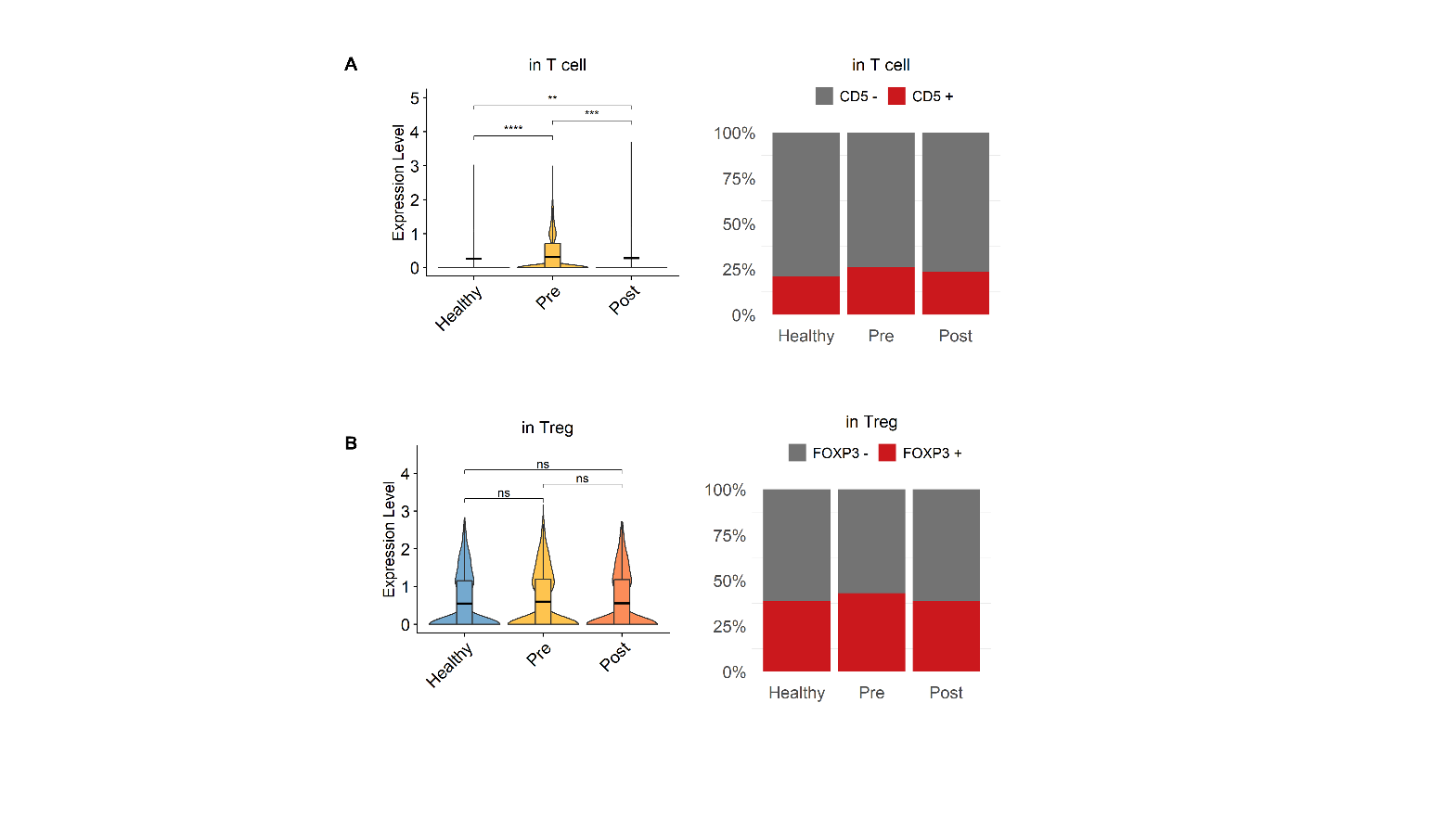


Fig. S14 Induction of immune tolerance cells and increase in Treg cells.

A. Expression of CD5 and proportion of CD5^+^ T cells in healthy, pre-, and post- treatment groups.

B. Expression of FOXP3 and proportion of FOXP3^+^ Treg cells in healthy, pre-, and post- treatment groups.

**Additional file 19**

**
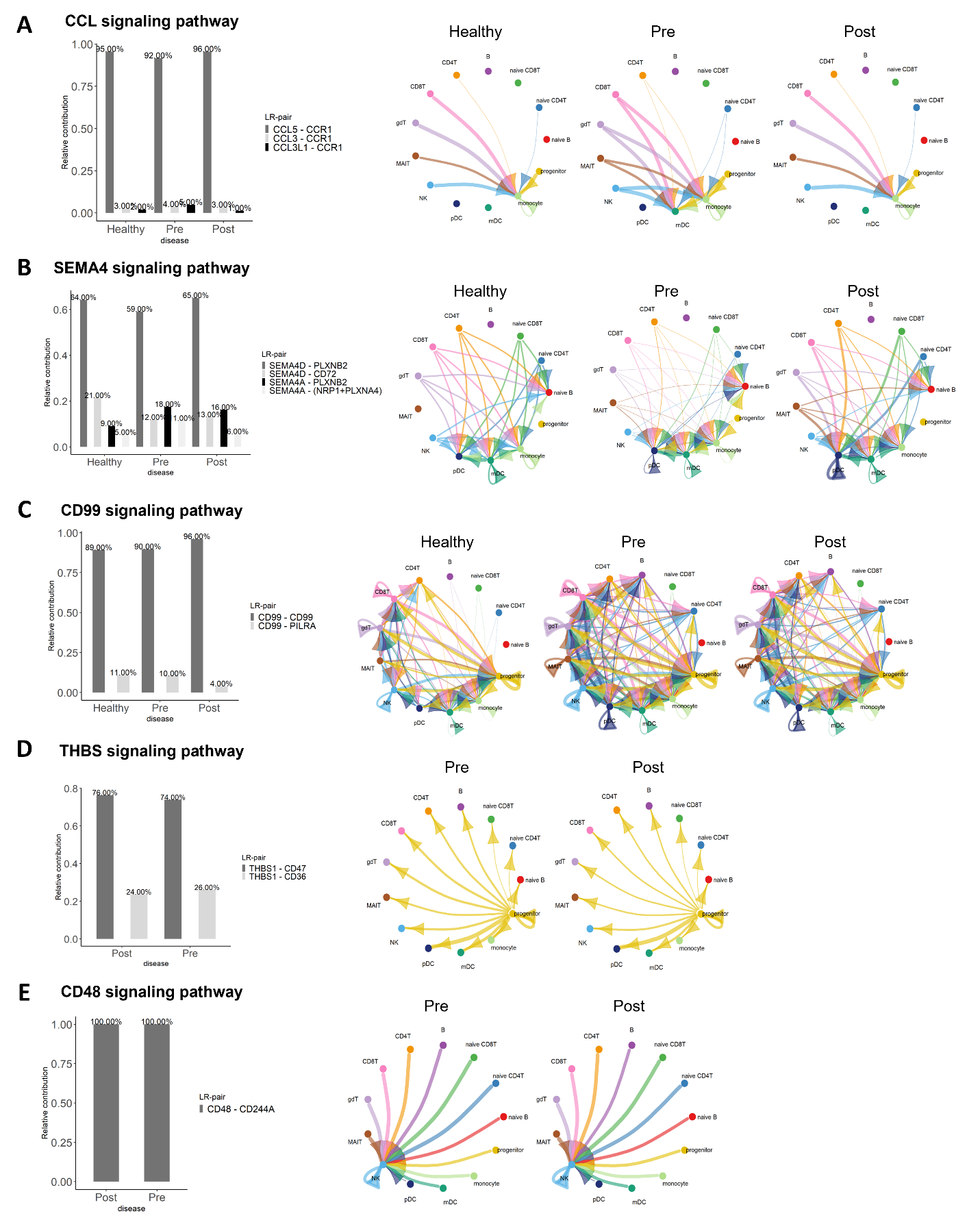
**

Fig. S15 The relative ligand‒receptor pair contribution and cellular network

Left panels are relative contribution plots of ligand-receptor pairs, and right panels are circle plots for CCL **(A)**, SEMA4 **(B)**, CD99 **(C)**, THBS **(D)**, and CD48 **(E)** signaling pathways. THBS and CD48 signals were not detected in the healthy group.
