# Supplementary material for: Single-cell RNA sequencing reveals rebalancing of immunological response in patients with periodontitis after non-surgical periodontal therapy
Source: J Transl Med. 2022 Nov 3;20:504. doi: 10.1186/s12967-022-03702-2 (PMC9635198; doi:10.1186/s12967-022-03702-2)
Supplement: Supplementary file 2 — Supplementary Material 2 [file 12967_2022_3702_MOESM2_ESM.docx]

*Journal of Translational Medicine*

Oct 5, 2022

Dear Francesco M. Marincola

I would like to thank you for your kind recommendation to submit our revised manuscript entitled “**Single-cell RNA sequencing reveals rebalancing of immunological response in patients with periodontitis after non-surgical periodontal therapy.**”

We have carefully considered all the thoughtful comments of the reviewers and revised the entire manuscript, including the title, to better reflect our results and conclusions. Specific responses to the reviewers’ comments are provided below. Please find the enclosed revised manuscript. We sincerely hope that these revisions will make the manuscript fit for publication in the *Journal of Translational Medicine*.

Thank you again for your comments and encouragement.

Sincerely yours,

Yun Hak Kim, M.D., Ph.D.

Department of Anatomy and Department of Biomedical Informatics

Pusan National University School of Medicine

49 Busandaehak-ro, Yangsan, 50612, Republic of Korea

Tel: +82-51-510-8091

Dear Reviewer 1:

**1. “This manuscript provides a single-cell RNAseq characterization of PBMCs in healthy patients and in patients with periodontal disease pre-and post- periodontal treatment. The manuscript is commended for providing a valuable data set that can be utilized by those in the fields of immunology and periodontal disease research. Basic computational analysis of the data set is completed in the manuscript to provide a general understanding of immune cell transcriptional differences across the three groups. This paper is interesting but has some major shortcomings that should be addressed as noted below. In addition, the manuscript would benefit greatly from language editing services of some type. It is appreciated that the manuscript may not be written in the authors' first language; however, there are grammatical and contextual errors that detract from the manuscript and make it difficult to fully interpret the findings.”**

🡪 Thank you for your kind comments. Based on your suggestions, we have revised the entire manuscript accordingly. Please consider the revised version of the manuscript.

**2. “(Introduction) Line 69-70: It is unclear why the authors mention bacteria of endodontic origin in the discussion about periodontal disease induced systemic inflammation.”**

🡪 We agree that mentioning bacteria of endodontic origin could be misleading. Accordingly, we have rewritten the sentences explaining the pathogenesis of periodontitis and modulation of the host immune response (line 65-72 on page 5). Thank you for your thoughtful comment.

**3. “(Introduction) Line 72-77: The discussion on mechanisms in which oral bacteria are pathogenic in systemic disease is too generalized. A more in depth discussion should be made here as it is pertinent to the main points of this manuscript.”**

🡪 We attempted to more clearly suggest a mechanism for the correlation between periodontitis and systemic disease by explaining the possible pathways of these diseases (line 73-84 on page 5). Thank you for the advice that helped to impart the logic of our research.

**4. “(Introduction) Line 80-83: The explanation of treatment modalities is not sufficient for an audience outside the dental field. There is no discussion of what non-surgical therapy is, what is meant by ‘cause-related therapy’, or why its considered the ‘gold standard’.”**

🡪 This study aimed to evaluate the transcriptional changes in immune cells before and after periodontal therapy. The paragraph explaining periodontal therapy seems to obscure our point, as periodontal treatment is not an important aspect. Therefore, we eliminated redundant discussion on periodontal therapy and focused on the goals of our study.

**5. “(Methods) Line 116: The exclusion criteria for tobacco use needs to be better defined. Only heavy smokers were excluded? Describe how heavy smoking is defined. If they were not heavy smokers but still used tobacco, were they included in the study?”**

🡪 We apologize for this lack of information. All participants were non-smokers or non-heavy smokers, which we defined as people who smoked less than one pack per day. The exclusion criteria were revised to supplement more details (line 110 on page 7).

**6. “(Methods) Table S1: the demographic data is not clearly presented. 12 total patients are presented in the table, but 8 patients are described in the text. 8 male and 8 female patients are listed in the table for a total of 16 patients. It is not clear if the four patients in the pretreatment group were the same as the four patients in the post-treatment group.”**

🡪 We apologize for the confusion and have revised the Table S1. The dataset used in this study consisted of four control samples from healthy donors, four samples from pre-treatment patients, and four samples from post-treatment patients. The pre- and post-treatment samples were paired samples from the same individuals. In the results section (line 229-231 on page 13), “four healthy donors and four paired pre- and post-treatment patients” are stated, and hopefully, this will not confuse the readers.

**7. “(Methods) Line 136-146: No inclusions criteria is reported for the subjects that donated blood for ELISA analysis. The first paragraph of the method section describes the inclusion criteria for subjects in the study but appears to only describe the 8 subjects used for PBMC collection. Were the subjects enrolled in the study different for ELISA compared to PBMC analysis? Did enrollment for both groups fall under the same IRB approval?”**

🡪 Thank you for pointing out this omission. The same inclusion criteria were applied for both the single-cell analysis and ELISA. We have added an explanation of the inclusion criteria and respective IRB approval, as well as the process of blood collection for ELISA (line 97, 100-102 on page 7, line 122, 129-130 on page 8).

**8. “(Results) Line 231-233: list the gene markers used to define each cell identity. In figure 1D, it is not clear what population is defined by every gene. Some genes appear to define no cluster such as IL3RA or ITGA2B. This may be a problem with the resolution and size of the image as it was necessary to zoom in to 300% to better see the images. Reformatting Figure 1 with larger images and text size would improve the readability of the figure.”**

🡪 We apologize for this and reformatted Figure 1. The cell types expressing IL3RA and ITGA2B were difficult to discern, as they were very few in number in the PBMC population. We have changed the structure of the figure and enlarged it as much as possible. The modified image is provided below.


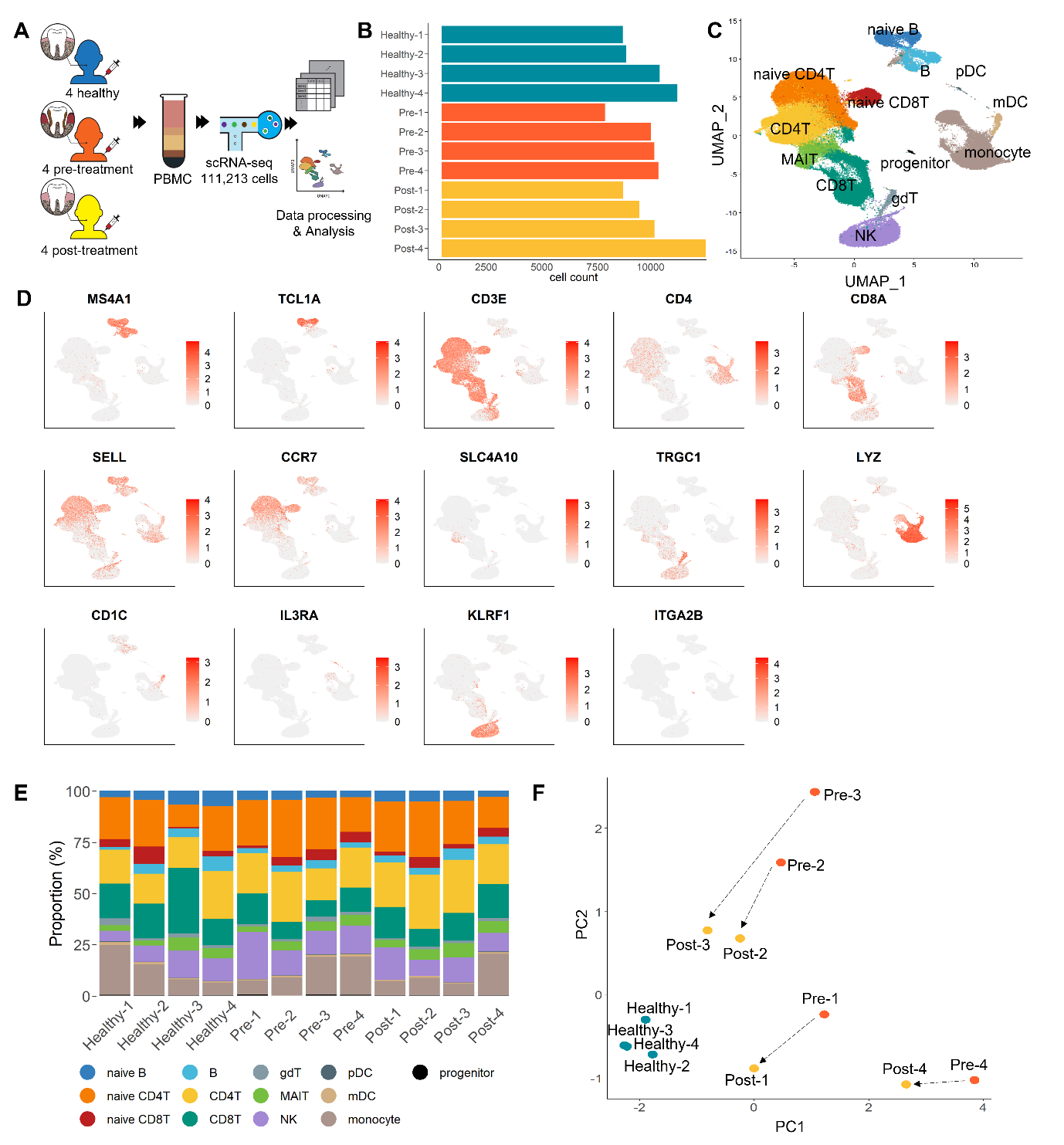


**9. “(Results) Line 242-249: The PCA analysis is described as utilizing 6 variables. It would be helpful to see the data from those 6 variables and how they change across the three groups. The authors conclude here that therapeutic intervention improves inflammatory status and clinical parameters but we can't see which specific parameters were actually changed when only the PCA plot is presented.”**

🡪 To show the integrative effect of treatment, we portrayed it using PCA, but it would also be informative to compare the individual clinical parameter as you have stated. The measurements for each variable are shown below.


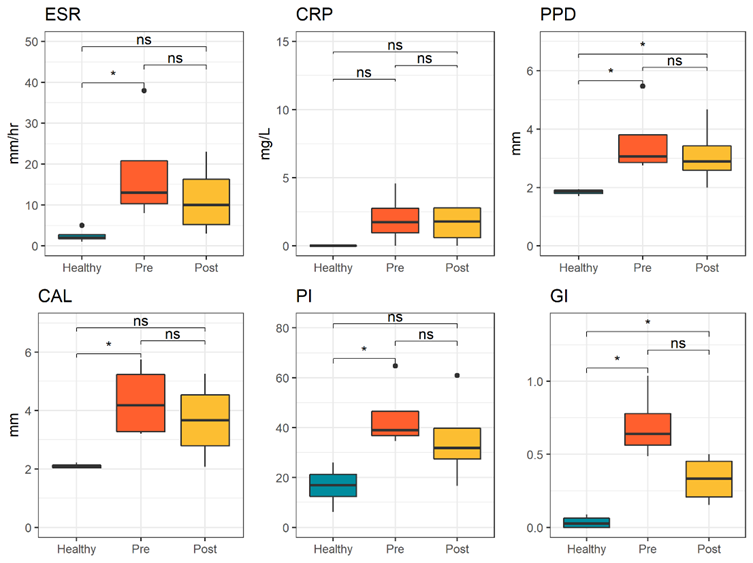


Although the clinical values of post-treatment patients were not perfectly rescued to those of the healthy controls and the differences between the pre- and post-treatment groups were not significant, we observed that all variables of the post-treatment patients were decreased compared to those of the pre-treatment patients, except for the CRP level. In light of these observations and the fact that the inflammation marker CRP gradually and continuously increases in chronic inflammation (<https://doi.org/10.1016/j.clinthera.2019.02.001>), we concluded that the reason for the incomplete rescue was that all patients had chronic periodontitis.


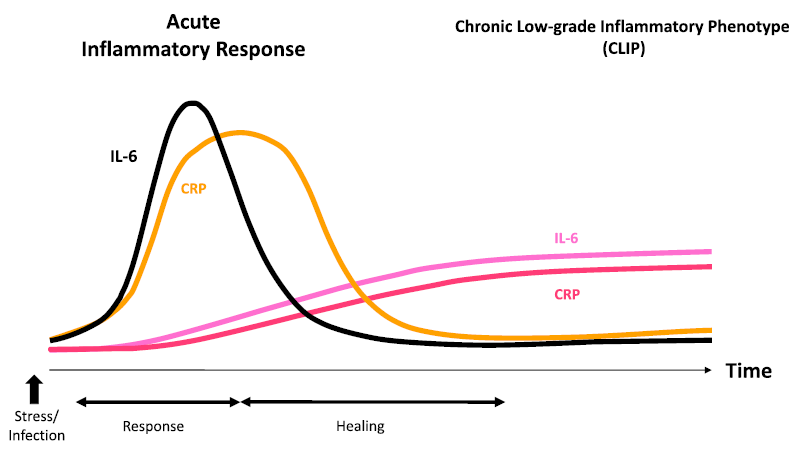


Thus, we have included this information in Additional file 5: Fig. S2 and Additional file 1: Table S1 and revised manuscript in line 248-252 on page 13-14. Thank you for your comment to help better understand our cohort and results.

**10. “(Results) Figure 2 and 3: how were the umaps of monocyte, B cell, and T cell subtypes generated? Were the cells identified as in figure 1 and then reanalyzed and reclustered? The clusters in Fig. 2A and Fig. 3 A, B, C do not appear to be the same as the corresponding cluster of cells depicted in Fig. 1C. If reclustering and reanalysis are performed after the initial analysis, this needs to be better explained in the Methods and Results section. Provide citations for the markers used to identify the cell subtypes identified in Fig. 2 and 3.”**

🡪 The cells in Fig. 1C were re-clustered and shown in Fig. 2A and Fig. 3A‒C. This seems to have cause confusion, as we did not provide a sufficient explanation of how this was accomplished. As you have suggested, we added the descriptions of sub-clustering for each paragraph and mentioned the sub-cluster markers to make it stand out (line 259-262 on page 14, line 278-280, 285-286 on page 15), including enlarging Additional file 6: Fig. S3.

**11. “(Results) Line 289-290: Are there any statistics you can provide to support the claim that the 4 genes of interest demonstrate increased expression in the pre-treatment group within the single cell RNAseq data set?”**

🡪 We identified genes which showed significantly higher expression in the pre-treatment group compared to both the healthy and the post-treatment groups, using FDR-adjusted p-values < 0.05. Those four genes satisfied this criterion. We selected these four genes based on the number of cells and cell types that changed their expression of those genes, rather than conducting additional statistical tests. Consequently, in more than half of the total cells, these genes were upregulated only in pre-treatment patients. Based on your comment, we have added supplementary information that provides readers with as much quantitative data as possible. This has been inserted in line 303-304 on page 16 of the main document and Additional file 14: Table S4.

**12. “(Results) Line 299-301: It is not clear how this conclusion was made from the data in Fig.S10. More explanation needs to be provided to support this conclusion.”**

🡪 We recognize the insufficient description of the figure you specified and that there was a statement that may have caused a misunderstanding. To address this, we repositioned the sentence in line 304-311 on page 16 and revised the statement “describe the improvements of relationship between –” into “describe the relationship –”. We also wrote additional interpretations to help us understand this figure. Thank you for helping us realize this insufficiency and improve the paper.

**13. “(Results) Line 306-310: Was there anything significant or important realized from the cell-cell interaction analysis? There is no description of what was found other than reference to the supplementary figure. Additional, discussion of the notable findings should be added here or in the discussion section.”**

🡪 We appreciate your comment that further clarification of the interaction list is required. We have described other significantly detected signal pathways from Additional file 17: Fig. 13 and revised the result (line 328-330 on page 17, line 373-377 on page 19) and discussion (line 424-427 on page 21-22, line 440-446 on page 22) of the main document, with the addition of Additional file 19: Fig. S15. From your remarks, we discovered additional pathways that can play a reciprocal role to the previously explored signals, and this will present more supportive evidence for our results.

**14. “(Results) Figure 5D: What gene expression is being demonstrated in each group? Is it an average of the three genes listed in the description (IL1, IL12, IL23)? Further explanation is needed.”**

🡪 As you stated, we have explored the average expression levels of representative pro-inflammatory cytokines secreted by dendritic cells. We identified two more typical DC-secreted cytokines, TNF and IFN$\alpha$ (<https://pubmed.ncbi.nlm.nih.gov/18258476/>). However, the IFN$\alpha$ gene was not included in our data because it was removed in the quality control step, and we excluded it from the list. In conclusion, we have included four genes and applied them to figure 5D. Accordingly, we have added an additional explanation to the manuscript (line 350-352 on page 18, line 772 on page 40). Thank you for your interest in our results and for your detailed review.

**15. “(Results) Line 335-336: How is differential signal intensity demonstrated in the circle plots? Is it quantifiable?”**

🡪 The applied analysis package ‘CellChat’ infers the intercellular communication probability using a statistical model called the Hill function to obtain the fraction of ligand binding receptor. Because the signal intensity is calculated as the probability rather than an absolute value, only a relative comparison is possible. However, in the circle plot, the thickness of the arrow is used as a means of intensity comparison. A thicker arrow implies a stronger intensity of cell communication. In addition, we extracted statistically significant pathways with a signal intensity greater than 1.5-fold when comparing the two groups. We added supplementary information for each figure caption of the circle plot and wrote the Methods section (line 218-219 on page 12). We hope this helps readers understand this figure.

We sincerely hope that these revisions will make the manuscript fit for publication in the *Journal of Translational Medicine*.

Dear Reviewer 2:

**The manuscript describes various analysis of the transcriptional profiles of immune cells in healthy patients and patients affects by periodontitis before and after treatment at single cell resolution. The authors analyzed several cell types (monocytes, B-cells, T-cells, and dendritic cells) with the purpose of finding differentially expressed genes (DEG) that can help to uncover what are the main factors of chronic inflammation, which is characteristic of periodontitis, and if the periodontal treatment can effectively help the immune system to recover. scRNA-seq analysis shows numerous genes which present similar patterns of expression in healthy and post-treatment patients, underlying the effectiveness of the treatment. The authors also found some common DEG among the various cell types with similar pattern within the groups and they propose one of them as a possible candidate to detect systemic inflammation during periodontitis. Furthermore, they also find two pathways which were rescued by treatment and one which was not, suggesting it as a possible therapeutic target in the treatment of periodontitis.**

**The work is per se interesting, as the importance of periodontitis and the necessity of finding alternative and valid new therapies. Though, there are several issues that need to be clarified:**

**1. “The authors should indicate the age of the patients enrolled and used in this study. Since the study is evaluating chronic inflammation, which is known to increase during aging, the age of the patients is a bias that must be taken in consideration.”**

🡪 We agree with your comments that it would be better if the age of the groups was completely matched. We suggest that the average age of healthy controls and periodontitis patients in Additional file 1: Table S1 and the average age of healthy donors and patients were 44 and 50, respectively. Though the healthy individuals also are in the age ranges with a high risk of periodontitis, we acknowledged the importance of age and added this limitation in the discussion (line 447-452 on page 22-23).

**2. “Based on the materials and methods section is unclear if the PBMSc isolated from patients' blood and then frozen are the same used to performed scRNA-seq analysis. If not, please clarify. If yes, although there is possibility to perform scRNA-seq analysis on frozen cells, the authors should be aware of the biases to which such analysis is subjected in these conditions, especially in analyzing chronic inflammation.”**

🡪 Both PBMC cell lines for scRNA-seq were frozen before sequencing and supplemented in the Methods section (line 128-130 on page 8). In this study design, there was a possibility that the patient would not revisit after treatment, so we had to sequence the frozen samples before and after treatment. Although many studies analyzing scRNAseq of PBMC frequently utilize frozen samples (<https://www.ncbi.nlm.nih.gov/pmc/articles/PMC8114807/>), there is a limitation since the viability of frozen PBMCs is lower than that of fresh ones, as you pointed out. To reduce batch effects and bias as much as possible, we sequenced only high-quality samples that passed the quality check. We have included this constraint in the discussion (line 453-457 on page 23), to help understand the limitations of our study.

**3. “Figure S3-S7 and S9: it is not clear to me what the authors are comparing in these figures. They should specify it as they have done in all the figure reporting DEG.”**

🡪 We are aware that we did not define the control group or the comparison group for supplementary data from your remark. Thank you for your feedback, which helped us to improve clarity, and we have corrected these matters in line 266-267 on page 14, line 287-289 on page 15, line 300-301 on page 16.

**4. “Dot plot data showing DEG among the three groups are interesting, as they clearly show similar patterns in the healthy and post-treatment group, and an opposite trend in the pre-treatment group. However, very few genes reported seems to be related with inflammation based on my knowledge. The authors should focus on showing only inflammation-related genes or better explain, even briefly, what are the genes that they are showing. Moreover, a confirm in the expression of at least few of such genes by other assays should be done.”**

🡪 As you discerned, there are not many genes or cytokines related to inflammation in PD. There were a few inflammatory genes such as CCL3L1 in NK cells, S100A12 in mDCs, TNFRSF1B in mDCs, and CCR7 in Th17 cells. However, from the ELISA results (revised Fig. 3G), the periodontitis patients were in a low-grade chronic inflammation state, since ESR showed a slight increase in chronic inflammatory status and CRP decreased after the resolution of acute inflammation (<https://www.ncbi.nlm.nih.gov/books/NBK333366/>). Therefore, only a few inflammation-related genes were extracted, because patients with periodontitis have a systemically weak inflammatory state. Thanks to your remarks, we have supplemented the interpretation of the observed inflammation-related genes in the Discussion section (line 395-405 on page 20-21). In addition, these results suggest that altered CRIP1 expression is an excellent marker for reflecting chronic inflammation induced by periodontitis. We appreciate your insightful remarks, which have helped us provide supportive evidence and highlight our results.

**5. “Figure 3: I wonder why the authors did not investigate about MIF, IFITM1, and RPS17 as well as they have done for CRISP1. It would have been very interesting.”**

🡪 As you can confirm in modified Additional file 14: Table S4, we focused on CRIP1, since it was a differentially expressed gene detected in many cell types, accounting for 96% of the total cells. As per your suggestion, we have gathered additional samples to clarify the level of IFITM1 in patients, which was the second most abundant cell type (approximately 71 %). The measured IFITM1 levels are provided in the figure below.


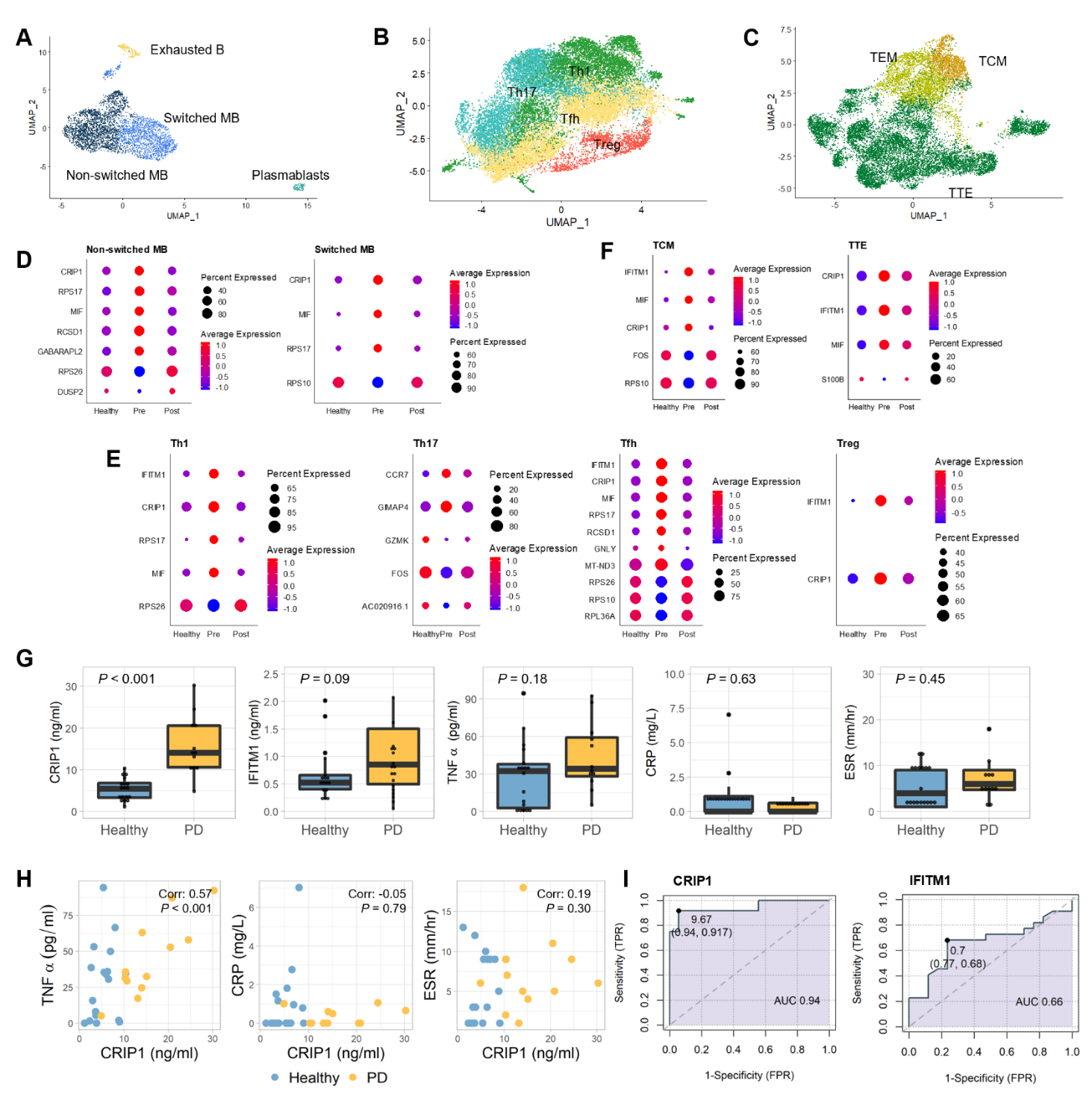


IFITM1 exhibited a similar pattern as CRIP1, although it was not as significant as CRIP1. This has been added in Fig. 3 and the Results section of the manuscript (line 312-321 on page 16-17). Further, we are conducting further research to investigate the aspect of remaining candidate markers *in vivo*, including CRIP1. Thank you for your careful suggestion. We were able to enhance the information and evidence of the results.

**6. “As a general comment, authors should avoid sentences like ‘…migration associate genes’, or ‘…gene associated with LPS’, etc. without specifying which genes are talking about or putting a figure as reference.”**

🡪 The use of ambiguous language could disrupt the flow and clarity of the article, but thanks to your suggestion, we were able to make improvements. We have corrected any ambiguous statements throughout the manuscript.

We sincerely hope that these revisions will make the manuscript fit for publication in the *Journal of Translational Medicine*.
